# Gut Microbe‐Driven Resistance Mechanisms in *Propylea Japonica*: Insights from Horizontal Gene Transfer and Oxidative Phosphorylation

**DOI:** 10.1002/advs.202520326

**Published:** 2025-12-12

**Authors:** Ningbo HuangFu, Xiangzhen Zhu, Zhijuan Tang, Li Wang, Kaixin Zhang, Dongyang Li, Jichao Ji, Jinjie Cui, Zhaojiang Guo, Junyu Luo, Xueke Gao

**Affiliations:** ^1^ Zhengzhou Research Base State Key Laboratory of Cotton Bio‐breeding and Integrated Utilization Institute of Cotton Research Chinese Academy of Agricultural Sciences Anyang Henan 455000 China; ^2^ College of Plant Science and Technology Huazhong Agricultural University Wuhan Hubei 430070 China; ^3^ Western Agricultural Research Center Chinese Academy of Agricultural Sciences Changji 831100 China; ^4^ National Key Laboratory of Cotton Bio‐breeding and Integrated Utilization Zhengzhou University Zhengzhou 450001 China; ^5^ College of Horticulture and Landscape Tianjin Agricultural University Tianjin 300392 China; ^6^ State Key Laboratory of Vegetable Biobreeding Department of Plant Protection Institute of Vegetables and Flowers Chinese Academy of Agricultural Sciences Beijing 100081 China

**Keywords:** acinetobacter, bacterial symbionts, dinotefuran, horizontal gene transfer, oxidative phosphorylation

## Abstract

Insect–microbial symbiont relationships are widespread in nature and often involve lateral gene transfer. Although the evolutionary processes that allow insects to adapt to complex environments remain largely unknown, it is clear that symbiotic relationships have essential roles in these processes. Here, gut microbes‐mediated regulation of *Propylea japonica* insecticide tolerance is found through modulation of a horizontally transferred gene (*P. japonica Domain unknow funcation 1, PjDUF1*) expression. However, this gene regulates the host capacity for dinotefuran tolerance by affecting the oxidative phosphorylation rate. This is confirmed by the RNAi‐Mediated Silencing of *PjDUF1*. Importantly, evidence is found that *PjDUF1* is donated from *Acenitobacter* via horizontal gene transfer (HGT). The findings provide the first experimental evidence that HGT events are important for pesticide tolerance in a prominent natural enemy species. Further study of the evolutionary origins of key natural enemy tolerance genes will shed additional light on how insects have developed resistance to adverse environments, suggesting strategies for protecting insect species that provide critical ecosystem services.

## Graphical abstract

*Acinetobacter* regulates dinotefuran tolerance in *Propylea japonica* by mediating the expression of the horizontally transferred gene *PjDUF1*.

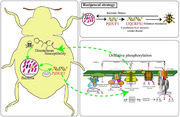

## 1 Introduction

Bacterial symbionts engage in interactions with their hosts, producing specialized metabolites that profoundly influence host reproduction, development, toxin resistance, and key nutrient provision.^[^ ^1^, ^2^, ^3^ ^]^ In return, bacterial symbionts rely on the host for nutrient acquisition and maintenance of a stable environment for parasitism. Large‐scale human intervention in the natural environment has led to unprecedented contamination levels with toxic compounds. Although insect evolution has a negligible effect in addressing the immediate survival pressures, bacterial symbionts can respond rapidly to environmental changes.^[^ ^4^, ^5^, ^6^ ^]^ Symbiotic bacteria can rapidly respond to changes in the environment in several ways: by altering population structure, by undergoing mutation, through the acquisition of novel microbes, or via horizontal gene transfer (HGT).^[^ ^7^ ^]^ Any of these strategies may provide the host with the necessary time to improve its fitness to cope with the harsh environment in the short term and to evolve in the long term.

HGT, also known as lateral gene transfer, provides a tremendous impetus for genomic tachytelic evolution in both prokaryotes and eukaryotes. However, the importance of HGT between prokaryotic and eukaryotic hosts or between eukaryotes is often overshadowed by the more frequent HGT events in prokaryotes.^[^ ^8^, ^9^ ^]^ Nevertheless, recent studies have indicated that far more HGT events have occurred among eukaryotes and between prokaryotes and eukaryotes than previously known. Among insects, ≈79.0% of HGT‐derived genes are donated from bacteria, 13.8% from fungi, 3.0% from plants, and 2.6% from viruses.^[^ ^10^, ^11^ ^]^ Some insect species are particularly amenable to gene acquisition via HGT from symbionts. For example, each known lepidopteran species has undergone at least 16 HGT events, and species in the order Hemiptera have acquired at least 13 genes each via HGT.^[^ ^10^ ^]^ Insect genes acquired via HGT have important growth, development, and survival roles. β‐fructofuranosidase, which plays a vital role in carbohydrate metabolism, is widespread among members of the order Lepidoptera, but is more sporadically present in hymenopteran and coleopteran species; the gene encoding it was horizontally transferred from bacteria.^[^ ^12^ ^]^ In an even more astonishing case, a horizontally transferred plant‐derived phenolic glucoside malonyltransferase gene allows whiteflies to efficiently neutralize phenolic glucosides, contributing to the pervasive host adaptability of whiteflies.^[^ ^13^ ^]^ Recent studies in *Riptortus pedestris* have demonstrated that the *Burkholderia* symbiont degrades the insecticide fenitrothion into a nontoxic compound (3‐methyl‐4‐nitrophenol) using a horizontally transferred gene encoding a detoxifying enzyme.^[^ ^4^ ^]^

Dinotefuran is a broad‐spectrum third‐generation neonicotinoid insecticide that is frequently used in agriculture. However, extensive use has resulted in high levels of dinotefuran residues in soil, water, various crops, and even the human bloodstream.^[^ ^14^, ^15^, ^16^, ^17^ ^]^ This compound disrupts the growth, development, and behavior of insect herbivores and profoundly affects predatory insects.^[^ ^18^ ^]^ *Propylea japonica* (Coleoptera: Coccinellidae) is an excellent natural enemy of aphids, whiteflies, and lepidopteran larvae (Figure S1, Supporting Information). Importantly, it exhibits remarkable tolerance to neonicotinoid insecticides.^[^ ^19^, ^20^ ^]^ Previous studies have found that insecticide resistance is likely associated with *Acinetobacter*.^[^ ^21^, ^22^ ^]^ For example, in *Aphis gossypii*, the abundance of *Acinetobacter* in neonicotinoid insecticide‐resistant strains was significantly higher than in sensitive strains.^[^ ^21^ ^]^ In addition, *Acinetobacter* promotes the development of insecticide resistance in *Aedes albopictus*.^[^ ^22^ ^]^ *Acinetobacter* can develop drug resistance through numerous mechanisms: enzymatic degradation of drugs, efflux pump activation, aminoglycoside modification, target site alteration, biofilm formation, and HGT.^[^ ^23^, ^24^, ^25^, ^26^ ^]^ In particular, HGT has become an important method for *Acinetobacter* to acquire and spread resistance genes rapidly.^[^ ^26^ ^]^ For instance, *A. baumannii* acquires cephalosporin resistance by Horizontal Transfer of an *ISAba125*‐activated *ampC* gene.^[^ ^27^ ^]^ *A. baylyi* can acquire an antibiotic resistance gene by HGT on Lettuce Phylloplane.^[^ ^28^ ^]^ *A. baylyi* spreads resistance genes to *Escherichia coli* through HGT.^[^ ^29^ ^]^ However, it is worth noting that these bacteria are very likely to spread resistance genes to insects and even some mammals. In particular, when these selective pressures are frequently used insecticides, these bacteria are highly likely to promote resistance development in host insects.

Here, using evolutionary analysis, molecular and biochemical approaches, and insect performance observations, we uncovered *Acinetobacter*‐mediated upregulation of a horizontally transferred gene (*PjDUF1*) expression contributing to *Propylea japonica* dinotefuran tolerance. The evolutionary origin of this gene was analyzed, as was the precise mechanism of action. Our study demonstrates for the first time a mutual regulatory relationship between symbiotic bacteria and host horizontally transferred genes; furthermore, it reveals an unexpected route by which *P. japonica* has evolved its extraordinary capacity for pesticide tolerance. This study enhances our understanding of the evolutionary pathways leading to insect stress tolerance and highlights the role of inter‐kingdom HGT in environmental adaptations.

## 2 Results

### 2.1 *Acinetobacter* Increased *P. Japonica* Insecticide Tolerance

**Figure 1:**
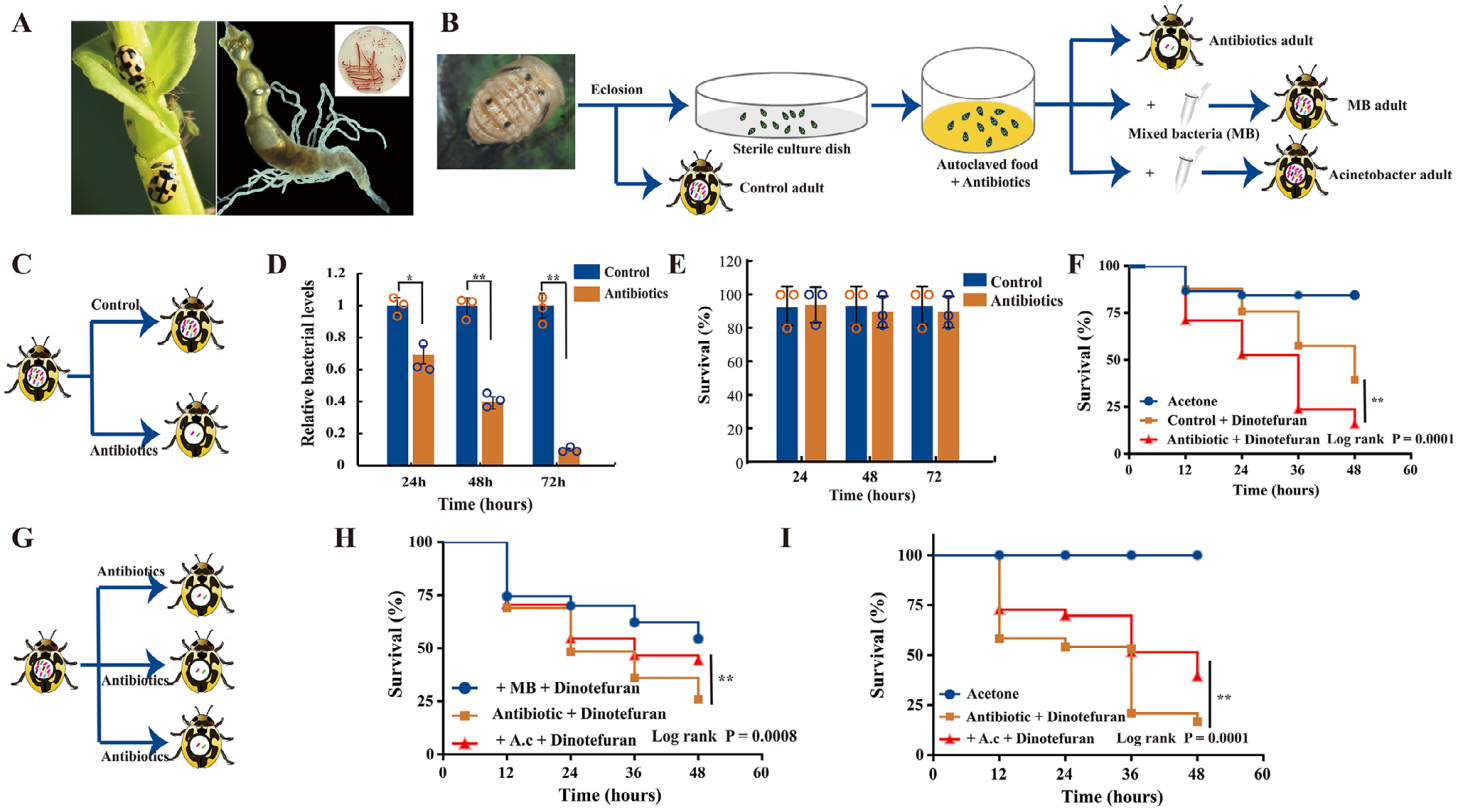
The *P. japonica* gut microbiome modulates dinotefuran susceptibility. A) Representative whole *P. japonica* adults (left) and a dissected gut (right). B) Schematic illustration of the workflow used to generate germ‐free (GF) and gnotobiotic *P. japonica*. C) Schematic illustration of the antibiotic treatment workflow. *P. japonica* was treated with an antibiotic cocktail and then assayed for bacterial content and insecticide susceptibility. D) Total bacterial quantification at 24, 48, and 72 h of continuous antibiotic treatment. E) Effects of antibiotic treatment on *P. japonica* survival rates at 24, 48, and 72 h. F) Effects of gut microbiome elimination on *P. japonica* dinotefuran susceptibility. Acetone, n = 33; control + dinotefuran, n = 33; antibiotic + dinotefuran, n = 38. G) Schematic illustration of the antibiotic treatment method. *P. japonica* was treated with an antibiotic cocktail and then assayed for insecticide susceptibility. H,I) *P. japonica* survival after dinotefuran treatment using topical application (H) or glass vial (I). For H, sample sizes were as follows: mixed bacteria (MB), n = 90; antibiotic + dinotefuran, n = 97; *Acinetobacter calcoaceticus* (*A.c*) + dinotefuran, n = 98. For I, sample sizes were as follows: acetone, n = 32; antibiotic, n = 24; *A. c*, n = 33. Statistical comparison was based on the log‐rank (Mantel–Cox) test, and the significance level for results was set at ^*^ *p* <0.05, ^**^ *p* < 0.

**Figure 2:**
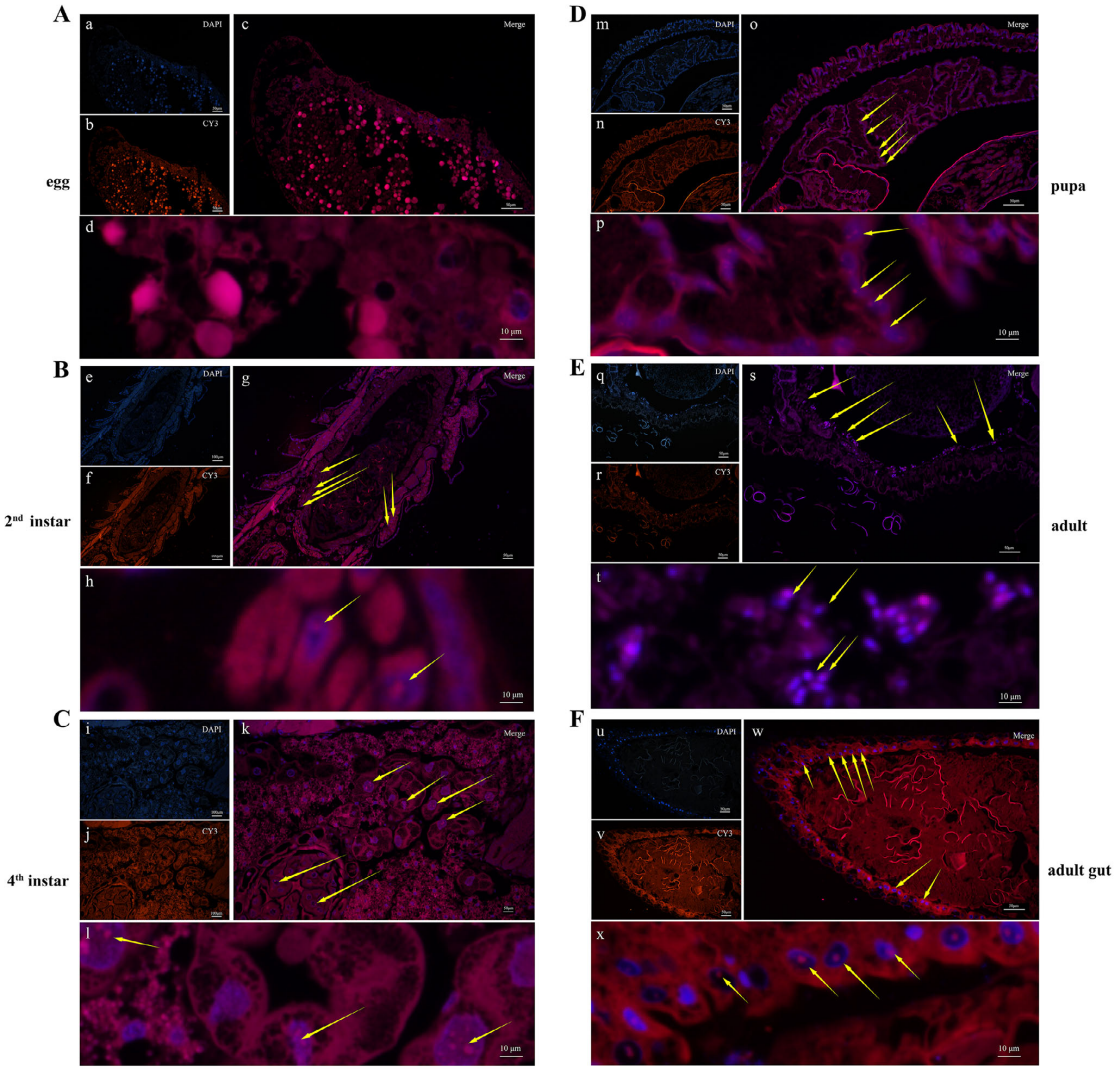
Localization of symbiotic *Acinetobacter* in *P. japonica* at different life stages.)A Whole‐ mount of a *P. japonica* egg showing no *Acinetobacter*. Sagittal sections of a second instar larva B) a fourth instar larva C) a pupa, and D)an adult E) showing dense *Acinetobacter* cultures in the gut (yellow arrows). F) Intestinal section showing the *Acinetobacter* population in the intestinal lining.

To determine whether *Acinetobacter* modulated *P. japonica* insecticide tolerance, first, we isolated *Acinetobacter* from the gut of *P. japonica* samples by a differential medium (**Figure** **1**A). In addition, we carried out fluorescence in situ hybridization (FISH) with specific probes targeting this strain. However, no *Acinetobacter* was detected in the eggs (**Figure** **2**A). *Acinetobacter* was successfully cultured from the guts of larvae, pupae, and adults (Figure 2B–E). These results suggested that ladybirds acquired gut‐colonizing *Acinetobacter* primarily from the environment (Figure S1B, Supporting Information). Subsequently, we performed an antibiotic treatment experiment; insects were treated with a cocktail of antibiotics that have previously been used to remove 90% of the gut bacteria of ladybirds (Figure 1A–D). PCR with 16S rDNA primers confirmed that the gut bacteria had been eliminated (Figure 1D). The antibiotic treatment alone did not significantly affect *P. japonica* mortality (Figure 1E), although *P. japonica* treated with antibiotics were more susceptible to dinotefuran; there was a higher mortality rate among these individuals after pesticide treatment than among control insects treated with pesticides (Figure 1F). However, supplementation with *A. calcoaceticus* significantly restored antibiotic‐treated *P. japonica* tolerance to dinotefuran (Figure 1G–I). *Acinetobacter* appears to have a propensity to acquire highly effective resistance mechanisms extremely rapidly.^[^ ^48^ ^]^ Previous studies have found that *Acinetobacter* is susceptible to gene exchange between microorganisms through HGT, so acquiring and spreading efficient resistance mechanisms is straightforward.^[^ ^48^, ^49^ ^]^ Therefore, we first examined whether *Acinetobacter* endows insecticide tolerance to host ladybirds via HGT.

### 2.2 The Identification of Putative Genes Derived from *Acinetobacter* in *P. Japonica*

**Figure 3:**
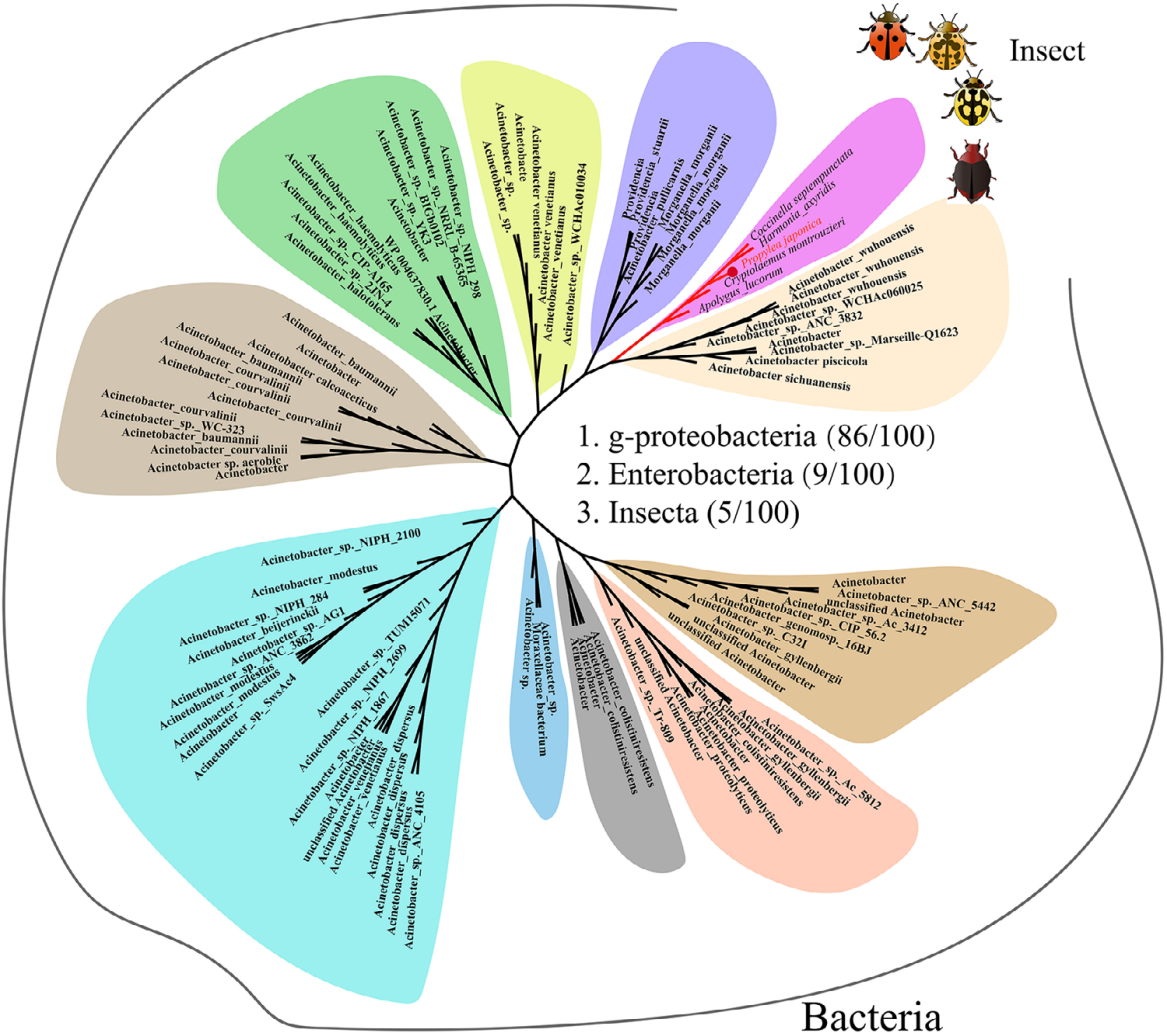
Phylogenetic analysis of *PjDUF1*. Evolutionary relationships were established using the maximum‐likelihood topology and the (LG+G+I+F) model with 1000 bootstrap replicates. Red and black branches indicate insects and bacteria, respectively.

To determine whether *Acinetobacter* endowed ladybirds with insecticide tolerance through HGT, we systematically identified genes in the *P. japonica* genome from bacteria. We found a total of 1757 genes in *P. japonica* genomes that were highly homologous to bacteria (with “Expect threshold” set at 1E‐40 and identity value≥30%) (Table S3, Supporting Information). We used a previously reported method^[^ ^33^ ^]^ to exclude vertically‐evolving and non‐bacterial origin genes from 1757 genes that are highly homologous to bacteria. We obtained 7 candidate horizontally transferred genes (HTGs) in *P. japonica* (Table S3, Supporting Information). These candidate HTGs and similar sequences were used to produce phylogenetic trees, but only one gene (*Pjao006920.1*, called *PjDUF1*) was highly homologous to *Acinetobacter* (**Figure** **3**).

### 2.3 *PjDUF1* Identification and Characterization

*PjDUF1*, which consisted of one exon, was cloned from adult *P. japonica* (Table S1, Supporting Information). The CDS was 774 nucleotides in length, encoding a protein of 257 amino acids. A BLASTP search against the predicted *P. japonica* proteome did not reveal the presence of a paralog. To understand the evolutionary history and phylogenetic relationships of insect *DUF1* genes, we systematically examined the distribution among high‐quality insect genomes using *PjDUF1* as the query. We found that the homologous genes identified were only distributed in Coleoptera, except for one in a hemipteran species (*Apolygus lucorum*) (Figure 3). Interestingly, these genes appear to be mainly restricted to predatory insects of the Coccinellidae (Figure S3A, Supporting Information). For example, there was one homolog in *Coccinella septempunctata* (*Csep019231.1*), two homologs in *Harmonia axyridis* (*XP_045463864.1*, *XP_045463866.1*), and three homologs in *Cryptolaemus montrouzieri* (*Cmon006612.1*, *Cmon006614.1*, *Cmon006615.1*) (Figure S3A, Supporting Information). The results suggest that the HGT event was conservatively estimated to have occurred in the common ancestor of Coccinellidae ≈203 million years ago. In addition, *PjDUF1* contained a conserved domain of unknown function (the DUF1963 domain) (Figure S4, Supporting Information).

### 2.4 Evidence for Horizontal Transfer of *PjDUF1* from *Acinetobacter* to Insects

A search of *PjDUF1* in InterProScan revealed that the DUF1963 domain is mainly found in bacteria; 6381, 13, and 130 protein sequences containing the DUF1963 structural domain were found in bacteria, archaea, and eukaryotes, respectively (Figure S5, Supporting Information). Furtherly, a BLASTP search against the NCBI NR database revealed that the closest *PjDUF1* homologs were present in bacteria (E‐value <2 × 10^−59^, bit‐score >200), except for those in the insects noted above. Surprisingly, most of the bacteria with genes closely related to *PjDUF1* were in the genus *Acinetobacter* (78/86) of the class g‐Proteobacteria (86/100) (Figure 3). Although it is not clear how the HTG of ladybirds originated from *Acinetobacter*, phylogenetic analysis suggests that the *PjDUF1* gene in the *P. japonica* and *Acinetobacter* share a co‐evolutionary history.

An independent genomic analysis was performed to confirm that *PjDUF1* was integrated into the *P. japonica* genome (Figure S6, Supporting Information). *PjDUF1* (*Pjao006920.1*) was found to be located on chromosome (Chr) 3 (CM024241.1), surrounded by genes encoding an uncharacterized protein (*Pjao008185.1*) on one side and an autophagy‐related protein (*Pjao006406.1*) on the other side (Figure S6A, Supporting Information). The two homologs in *H. axyridis* (*XP_045463864.1* and *XP_045463866.1*) were located on Chr2 (*OU611928.1*), with genes encoding an uncharacterized protein (XP_045462205.1) on one side and a surfeit locus protein (XP_045462215.1) on the other. In *C. montrouzieri*, the three homologous genes (*Cmon006612.1*, *Cmon006614.1*, and *Cmon006615.1*) were located on contig JABFTP010000352.1 and were surrounded by genes encoding the uncharacterized proteins Cmon006613.1 and Cmon006616.1 (Figure S6A, Supporting Information). In addition, the Synteny analysis showed that the *PjDUF1* genes and its surrounding genes in the closely related species of *P. japonica* exhibit high conservation (Figure S6B, Supporting Information). In *P. japonica*, PCR amplification and Sanger sequencing confirmed that *PjDUF1* was inserted into the genes encoding the uncharacterized protein (Pjao008185.1) (Figure S6C–E, Supporting Information). This confirmed the presence of the HGT‐derived gene within the *P. japonica* genome.

### 2.5 Spatio‐Temporal *PjDUF1* Expression

Spatio‐temporal *PjDUF1* expression patterns were determined in *P. japonica* with qRT‐PCR at six developmental stages (first instar larvae, second instar larvae, third instar larvae, fourth instar larvae, pupae, and adults at 1–3 d after emergence) and in several regions of the adults (the leg, elytra, midgut, abdomen, head, and thorax). The results showed that *PjDUF1* was expressed at all developmental stages and tissues (Figure S7, Supporting Information). The significantly higher expression in male adults suggests that *PjDUF1* plays its most important role in the male adult stage. Moreover, *PjDUF1* was most highly expressed in the leg and abdomen, which indicates that it is likely to play an important role in the biological functions related to the legs and abdomen (Figure S7, Supporting Information).

### 2.6 RNAi Knockdown of the *PjDUF1* Gene Reduces *P. Japonica* Insecticide Tolerance

**Figure 4:**
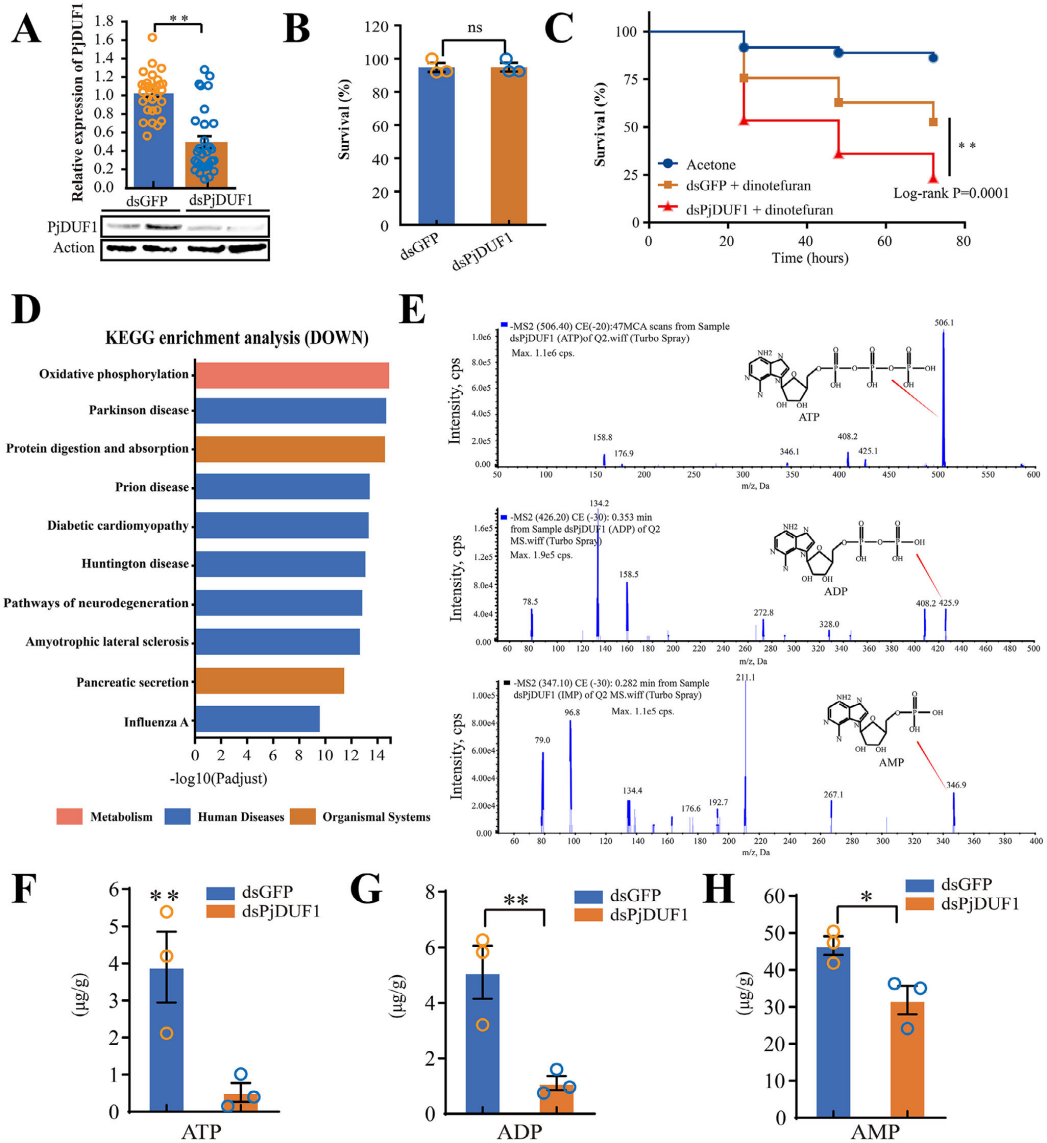
*PjDUF1* regulated host insect dinotefuran tolerance through oxidative phosphorylation signaling. A) *PjDUF1* expression after *PjDUF1*‐dsRNA injection. ^*^ *p* <0.05. n = 6. B) Effects of *PjDUF1‐*dsRNA treatment on insect survival. Ns, no significant difference. n = 3. C) *P. japonica* survival after *PjDUF1‐*dsRNA treatment followed by dinotefuran treatment (topical application). Acetone control, n = 38; dsGFP + dinotefuran, n = 78; dsPjDUF1 + dinotefuran, n = 86. Statistical comparison was based on the log‐rank (Mantel–Cox) test, ^*^ *p* <0.05, ^**^ *p* < 0.01. D) Effects of *PjDUF1* silencing on the oxidative phosphorylation signaling pathway. Blue boxes represent significantly downregulated DEGs. E) The ATP, ADP, and AMP levels were examined by UPLC‐MSMS. Effects of *PjDUF1* silencing on F) ATP, G) ADP, and H) AMP levels of *P. japonica*.

To investigate whether *Acinetobacter* endows insecticide tolerance to host ladybirds by the *PjDUF1* gene. RNAi was next applied to silence *PjDUF1* expression. Treatment with *PjDUF1*‐dsRNA successfully decreased *PjDUF1* expression by 65% after 2 d (**Figure** **4**A). *PjDUF1* silencing did not significantly affect *P. japonica* mortality rates compared to untreated controls (Figure 4B). However, dinotefuran tolerance was significantly decreased among *P. japonica* with *PjDUF1* silenced (Figure 4C).

### 2.7 *PjDUF1* Regulated Ladybird Insecticide Tolerance Through Mediating Oxidative Phosphorylation Signaling

To confirm whether the *PjDUF1* is directly able to metabolize pesticides, PjDUF1 was heterologously expressed in *Spodoptera frugiperda* (Sf9) cells for in vitro enzyme activity tests. The results showed that the heterologously expressed PjDUF1 protein cannot directly metabolize dinotefuran (Figure S8, Supporting Information). To further investigate the molecular mechanism by which *PjDUF1* mediated increased dinotefuran tolerance, RNA‐seq data from control and *PjDUF1*‐treated insects were analyzed, and DEGs were annotated. Oxidative phosphorylation was the most significantly downregulated signaling pathway among insects with *PjDUF1* knocked down (Figure 4D) (Table S4, Supporting Information). Specifically, knocking down *PjDUF1* significantly reduced the expression of NADH dehydrogenase (*NDUFS3, NDUFS6, NDUFA7, NDUFA13, NDUFV1, NDUFB3, NDUFA1, NDUFB7, NDUFA2, NDUFB9, NDUFA4, NDUFB10, NDUFA5, NDUFC2, NDUFA6*, and *NDUFC2*), succinate dehydrogenase (*SDHC*, *SDHD‐1*, and *SDHD‐2*), cytochrome c reductase (*UQCRFS1*, *QCR8‐1*, and *QCR8‐2*), cytochrome c oxidase (*COX4‐1*, *COX4‐2*, *COX5B*, *COX6A‐1*, *COX6A‐2*, *COX6B‐1*, *COX6B‐2*, *COX6C‐1*, *COX7A‐2*, *COX7A‐3*, and *COX7C*), and ATP synthase (*ATPeF0F‐1*, *ATPeF0F‐2*, *ATPeV0B, ATPeV0E, ATPeV1E, ATPeF0C, ATPeFG, ATPeV1F, ATPeF0B, ATPeV1F, ATPeF1B*, and *ATPeVS1*) genes (Figure S9 and Table S5, Supporting Information). Confirmatory qRT‐PCR experiments yielded similar results (Figure S10, Supporting Information).

To evaluate the impact of *PjDUF1* gene silencing on oxidative phosphorylation levels, we examined the ATP level by UPLC‐MSMS. Knocking down *PjDUF1* significantly reduced the 86.61% ATP level (Figure 4E,F). Additionally, the ADP and AMP levels were also significantly lower in dsPjDUF1 groups than in control groups (Figure 4G,H). These findings suggested that silencing *the PjDUF1* gene reduces oxidative phosphorylation levels and inhibits energy production.

**Figure 5:**
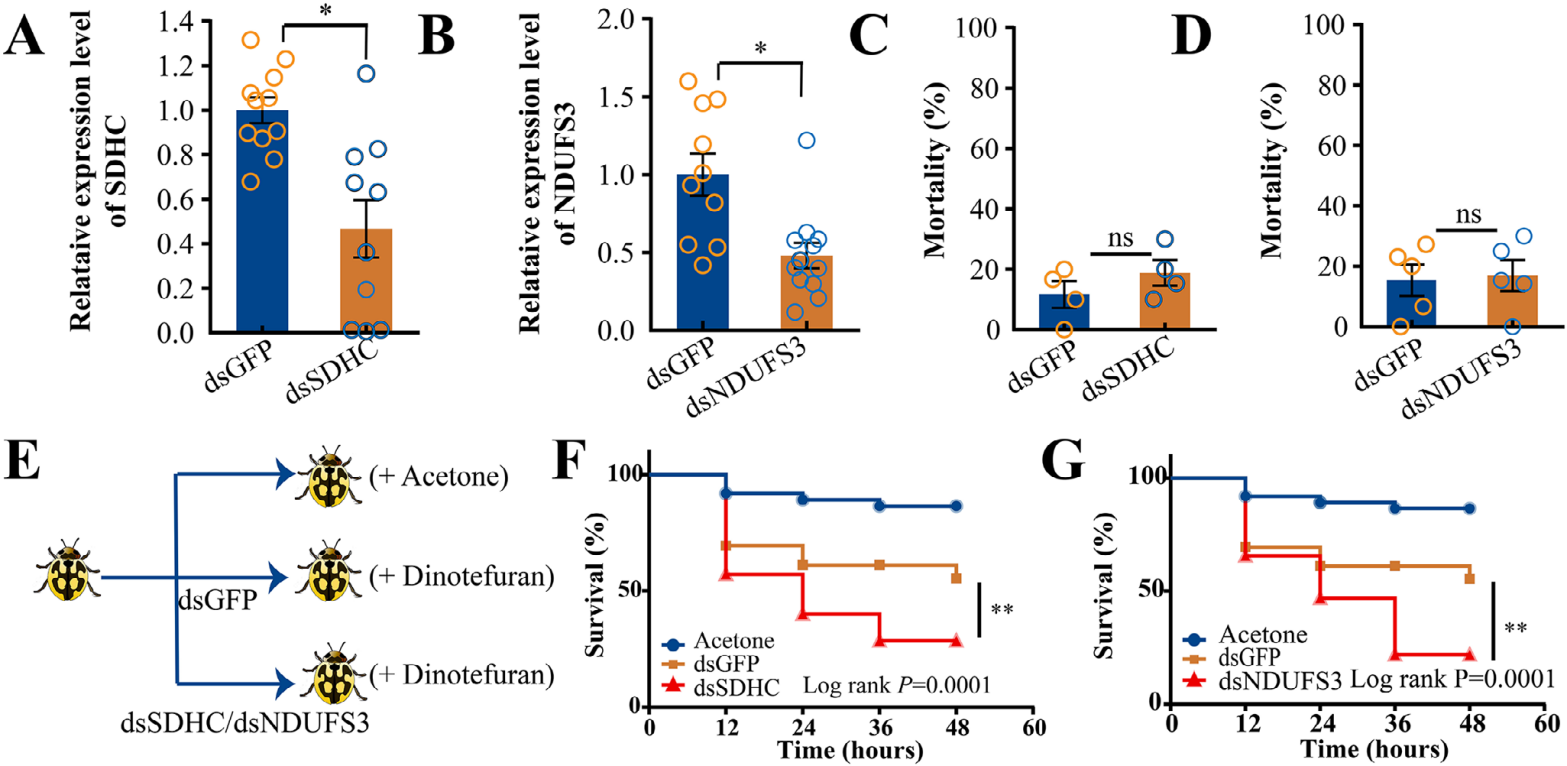
Effects of impaired oxidative phosphorylation on *P. japonica* dinotefuran tolerance. Relative expression levels of A) *SDHC* and B) *NDUFS3* after injection of dsRNA by qRT‐PCR. ^*^, *p* <0.05; ns, no significant difference. n = 6. Effects of C) *NDUFS3* and D) *SDHC* knockdown on *P. japonica* mortality. ns, no significant difference. n = 6. E) Design of the experiment to confirm the roles of *SDHC* and *NDUFS3* in regulating insecticide resistance. Survival curves of *P. japonica* in response to dinotefuran exposure after injection with F) dsSDHC or G) dsNDUFS3 Acetone, n = 37; dsGFP, n = 35; dsSDHC, n = 35; dsNDUFS3, n = 31. Statistical comparison was based on the log‐rank (Mantel–Cox) test, and the level of significance for results was set at ^*^ *p* <0.05 and ^**^ *p* <0.01.

To determine whether ladybird dinotefuran tolerance was related to oxidative phosphorylation level, oxidative phosphorylation was repressed by silencing two key genes. RNAi was used to inhibit the expression of genes encoding the succinate dehydrogenase complex (SDHC) (by 58%) and NADH dehydrogenase iron‐sulfur protein 3 (NDUFS3) (by 51%) (**Figure** **5**A,B). At 3 d post‐interference, mortality rates were consistent between insects with *SDHC* or *NDUF3* silenced and the dsGFP control (Figure 5C,D). As expected, knocking down *SDHC* or *NDUFS3* markedly decreased ladybird dinotefuran tolerance (Figure 5E–G). In addition, we further verified that the reduction in oxidative phosphorylation levels led to a decrease in the tolerance of ladybirds to pesticides by using oxidative phosphorylation inhibitors (Antimycin A) (Figure S11, Supporting Information). These results indicated that *PjDUF1* mediated dinotefuran tolerance in ladybirds by activating oxidative phosphorylation signaling.

### 2.8 *PjDUF1* Interacts with UQCRFS1 and Regulates Oxidative Phosphorylation Levels of Ladybirds

**Figure 6:**
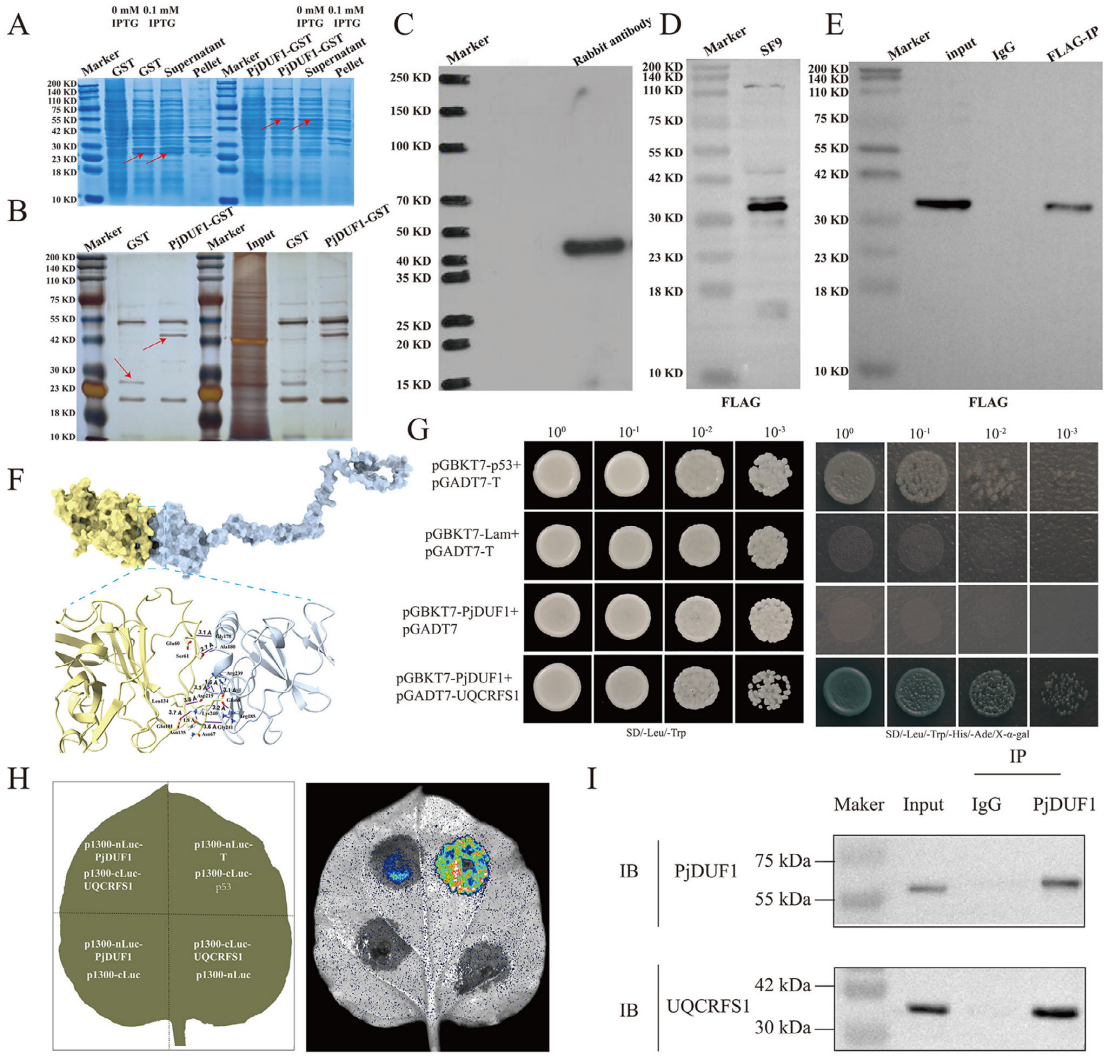
Identification of the interacting proteins of PjDUF1. A) Expression of recombinant GST‐PjDUF1. B) Identification of PjDUF1‐interacting proteins using GST pull‐down analysis. The gel was visualized using silver staining. C) Western blot analysis of antibodies against PjDUF1. D) The detection of FLAG label protein. FLAG label protein were purified using FLAG‐Tag Polyclonal Antibody (Rabbit) conjugated BeyoMag™ Protein A+G magnetic beads and separated with SDS‐PAGE. E) Western Blot results showed that FLAG label protein was detected in both input group and IP group, while no FLAG protein was detected in IgG group. F) Protein‐protein docking by AlphaFold3 and HADDOCK software. G) Analysis of PjDUF1 interaction with UQCRFS1 protein using yeast two‐hybrid system. The bait plasmid pGBKT7‐PjDUF1 and prey plasmid pGADT7‐UQCRFS1 were co‐transformed into the Y2HGold strain to confirm the protein‐protein interaction. pGBKT7‐p53 and pGADT7‐T co‐transformed Y2HGold cells served as a positive control, and pGBKT7‐Lam and pGADT7‐T co‐transformed Y2HGold cells were a negative control. H) Analysis of PjDUF1 interaction with UQCRFS1 protein by Luciferase Complementation Assay. The P1300‐nLUC‐T and P1300‐CLUC‐p53 were the positive control. I) The CO‐IP assay proved the interactions between PjDUF1 with UQCRFS1. IB, immunoblot.

To investigate how *PjDUF1* regulates oxidative phosphorylation levels, we used PjDUF1 as a bait to identify potential interacting proteins by GST Pull‐down/MS and CoIP/MS assays (**Figure** **6**A‐E). A total of 33 proteins were found to be PjDUF1‐interacting proteins (Table S6, Supporting Information). However, in the SF9 cells of CoIP/MS assays, 716 proteins were identified as potential PjDUF1‐interacting proteins (Table S7, Supporting Information). A total of five proteins, Proteasome subunit beta type‐6 (PSMβ6), Protein FAM76A, Integrator complex subunit 10 (INTS10), 60S ribosomal protein L15 (RpL15), and Cytochrome b‐c1 complex subunit Rieske (UQCRFS1), were identified using the two methods (Table S8, Supporting Information). Therefore, in follow‐up experiments, we focused on verifying these five proteins.

To verify the relationship between PjDUF1 and potential interacting proteins, we performed protein‐protein docking using AlphaFold3 and HADDOCK software. The docking results show a strong interaction between PjDUF1 and UQCRFS1, achieved through multiple amino acids (Figure 6F; Table S9, Supporting Information). To further verify the relationship between PjDUF1 and UQCRFS1, we performed yeast‐two‐hybrid (Y2H) assays to confirm the interaction between each pair of proteins. The results of Y2H assays indicated that PjDUF1 interacted with UQCRFS1 on SD medium lacking Ade, His, Leu, and Trp (Figure 6G). Additionally, luciferase complementation and CoIP assays were consistent with Y2H assays (Figure 6H,I). Collectively, these findings confirmed that PjDUF1 interacts with UQCRFS1 and regulates the oxidative phosphorylation levels.

### 2.9 The *PjDUF1* Gene is Involved in Regulating Athletic and Predatory Ability

**Figure 7:**
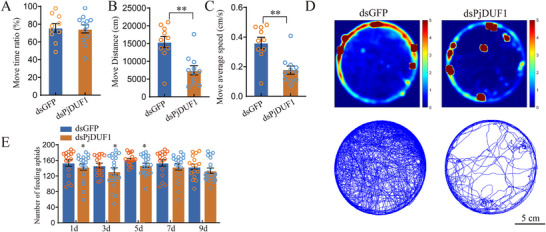
Effects of PjDUF1 silencing on the athletic and predatory abilities. The effect of *PjDUF1* silencing on the A) move time ratio B) move distance, and C) average speed. D) Example heatmap and tracks, 12 h from each insect. Scale bars, 5 cm. E The effect of *PjDUF1* silencing on Predation levels.

To verify that *PjDUF1* participates in regulating athletic and predatory abilities, we conducted RNAi experiments and found that *PjDUF1* silencing did not significantly impact the move time ratio compared to untreated controls (**Figure** **7**A). However, silencing of *PjDUF1* significantly reduced move distance and speed (Figure 7B,C, and D). Moreover, silencing of *PjDUF1* significantly reduced the number of aphids that ladybirds ate on days 1, 3, and 5 (Figure 7E), supporting the idea that *PjDUF1* affects the athletic and predatory abilities of ladybirds by regulating the level of oxidative phosphorylation. After silencing the *PjDUF1* gene, the ladybird's willingness to exercise did not weaken, but the energy generated was insufficient to maintain exercise needs.

### 2.10 Expression Levels of *PjDUF1* and the Abundance of *Acinetobacter* Influence Each Other

**Figure 8:**
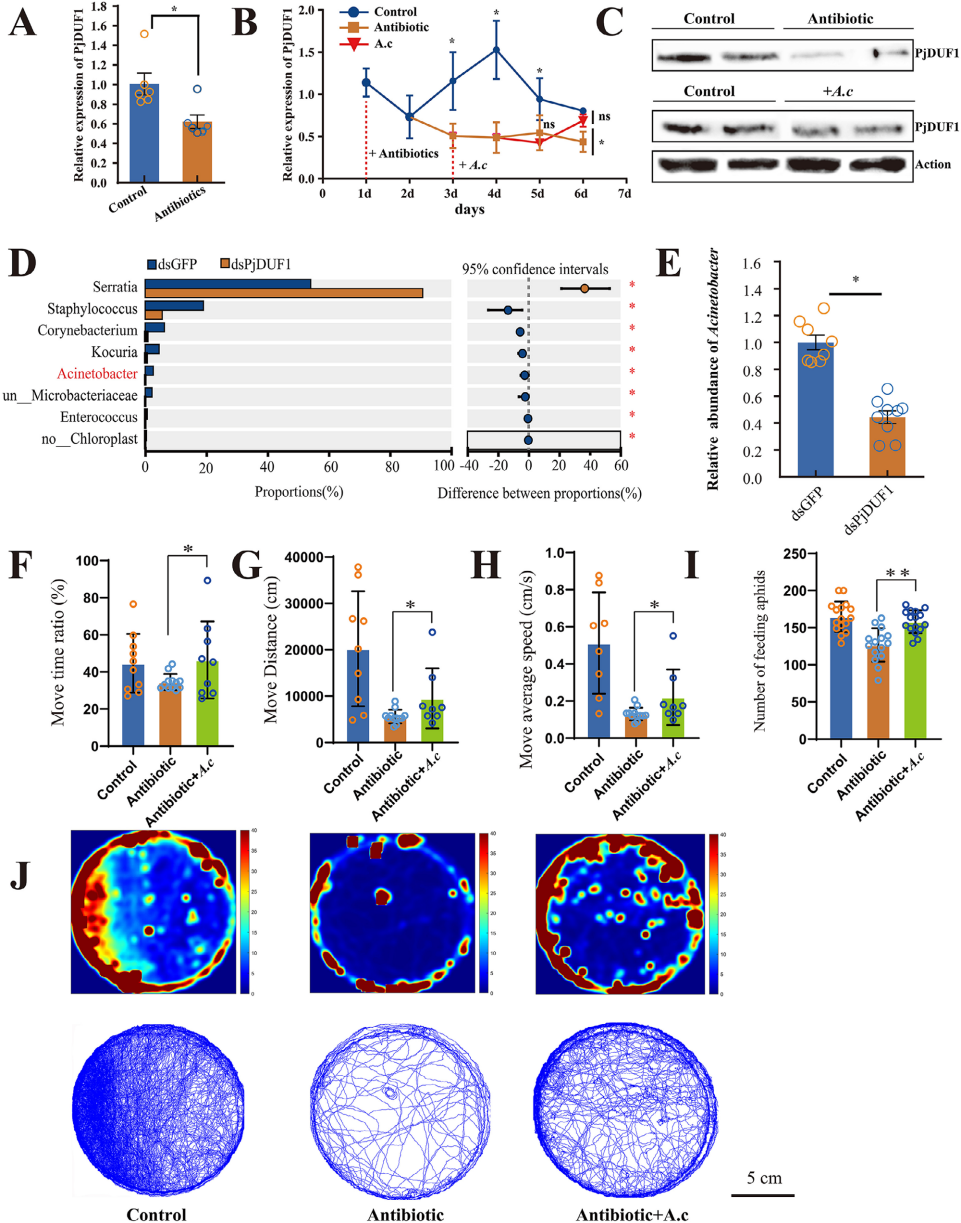
Effects of Acinetobacter on the Athletic and predatory ability. A) Effects of antibiotic treatment on *PjDUF1* expression. ^*^ *p* < 0.05, ^**^ *p* < 0.01. n = 6. B) Effects of *Acinetobacter* supplementation on *PjDUF1* expression. Control, standard feeding; antibiotic, continuous antibiotic treatment; *A. calcoaceticus* (*A. c*), supplementation with *Acinetobacter* after 3 d of antibiotic treatment. ^*^ *p* < 0.05, ^**^ *p* < 0.01. n = 6. C) The expression level of PjDUF1 proteins after *PjDUF1*‐dsRNA injection. ^*^ *p* < 0.05. n = 6. D) Effects of *PjDUF1* silencing on the abundance of microorganisms. E) The relative abundance of *Acinetobacter*. Data were means ± SE. ∗ represented the level of significance difference *p* < 0.05 in independent‐samples *t*‐test. Effects of *Acinetobacter* supplementation on the F) move time ratio, G) move distance, H) average speed, and I) Predation levels. J) Example heatmap and tracks, 12 h from each insect. Scale bars, 5 cm.

To verify whether *Acinetobacter* can directly regulate the life activities of host insects by influencing *PjDUF1*, a series of quantitative experiments were performed. We found that the expression of the *PjDUF1* gene was strongly correlated with the abundance of *Acinetobacter* (**Figure** **8**). Antibiotic cocktail treatment decreased *PjDUF1* expression by 45% (Figure 8A). To validate this result, *P. japonica* was administered an antibiotic cocktail, then supplemented with *Acinetobacter*. This result again showed that antibiotic treatment reduced *PjDUF1* expression significantly (by 46–69%). However, subsequent *Acinetobacter* supplementation significantly increased *PjDUF1* expression again (by 50%) compared to those treated only with antibiotics (Figure 8B). Western blotting analysis confirmed the interaction between *Acinetobacter* and *PjDUF1* gene. As shown in Figure 8C, the antibiotic cocktail treatment reduced *PjDUF1* expression, and *Acinetobacter* supplementation increased *PjDUF1* signal. We examined whether the expression level of *PjDUF1* can affect the abundance of *Acinetobacter* by 16S rRNA sequence (Table S10, Supporting Information). The 16S rRNA gene sequence analysis and qPCR demonstrated that the abundance of *Acinetobacter* decreased with the knockdown of *PjDUF1* (Figure 8D,E). These results indicated that the abundance of *Acinetobacter* was positively correlated with the expression level of *PjDUF1*, supporting the hypothesis that *Acinetobacter* increased its suitability in host insects by modulating *PjDUF1* expression.

While we have established that *Acinetobacter* abundance is tied to the expression level of *PjDUF1* and that *PjDUF1* plays a vital role in the energy production of host insects, we do not know the direct influence of *Acinetobacter* levels on the athletic and predatory abilities of ladybirds. Therefore, we examined the effects of *Acinetobacter* levels on the athletic and predation abilities of *P. japonica*. Supplementation of *Acinetobacter* significantly increased the move time ratio, move distance, average speed, and the number of predations (Figure 8F–I). The move distance increased by 69% compared to the antibiotic treatment group (Figure 8G,J).

#### 2.10.1 Discussion

The critical role of bacterial symbionts in assisting host insects in coping with various external stresses has been widely recognized.^[^ ^2^ ^]^ Bacterial symbionts promote host insect fitness in multiple insects by contributing to the host's digestion, nutrition, detoxification, and pathogen resistance.^[^ ^50^ ^]^ We found that *Acinetobacter* promoted host insecticide resistance through historical horizontal transfer of a bacterial gene to the host. This mechanism of increased host resistance to insecticides is achieved by regulating oxidative phosphorylation levels to increase host insect fitness. Moreover, changes in levels of oxidative phosphorylation mediated by the bacterial origin genes affect the locomotion and predation of host insects.

*Acinetobacter* is widely known for its ability to acquire antibiotic resistance rapidly.^[^ ^45^, ^53^ ^]^ It consists of at least 85 species (https://www.bacterio.net/genus/acinetobacter) in different environments, such as soil, water, and plants.^[^ ^52^ ^]^ Recent studies have shown that *Acinetobacter* is crucial in enhancing the host insect resistance to insecticide and external pathogens.^[^ ^22^, ^48^ ^]^ We have successfully isolated *A. calcoaceticus* from the gut of *P. japonica* samples. *A. calcoaceticus* is the most commonly identified species in the genus *Acinetobacter*, and it has a high frequency of natural transformation.^[^ ^49^ ^]^ This implies that *A. calcoaceticus* can easily exchange genes with the external environment. To date, the exchange of resistance genes between different species via horizontal gene transfer has been widely reported in *Acinetobacter*.^[^ ^51^, ^53^ ^]^ Therefore, we examined whether *A. calcoaceticus* endows ladybirds with insecticide resistance via horizontal gene transfer. Fortunately, a horizontally transferred gene in ladybirds was highly homologous to *Acinetobacter* following systematic identification.

RNAi confirmed that *PjDUF1* plays an essential role in dinotefuran resistance. Previous studies demonstrated that these HTGs are important in detoxification metabolism, nutrient absorption, and antibacterial.^[^ ^13^, ^43^, ^54^ ^]^ For instance, most lepidopteran HGT‐derived genes involve detoxification, metabolism, digestion, and adaptation.^[^ ^12^, ^33^ ^]^ In addition, recent studies in lepidopterans have shown that horizontally transferred genes are closely related to male courtship behavior.^[^ ^10^ ^]^ However, studies in Hemiptera have indicated that most horizontally transferred genes function in biotin provisioning, nutrient synthesis and antifungal.^[^ ^55^, ^56^, ^57^, ^58^, ^59^ ^]^ For example, whiteflies contain horizontally transferred biotin genes (*bioA*, *bioD*, and *bioB*), two bacteria‐derived *urea carboxylase* (*BtUCA*) and *allophanate hydrolase* (*BtAtzF*) genes, and aphids contain carotenogenic genes derived from fungi.^[^ ^55^, ^56^, ^57^ ^]^ Furthermore, some phytophagous coleopteran beetles contain HGT‐derived plant cell wall‐degrading enzyme genes (*PCWDEs*).^[^ ^54^ ^]^ For example, the glycoside hydrolase (GH) genes *GH28*, *GH32*, *GH43*, and *GH44* (in addition to *PL4*) were obtained through horizontal transfer from bacteria and fungi and are known to play important roles in lignocellulose degradation.^[^ ^60^ ^]^ However, in this study, we report for the first time that HTGs mediated *P. japonica* tolerance to the insecticide.

Further, *PjDUF1* regulated ladybird insecticide tolerance by mediating oxidative phosphorylation. RNA‐seq results showed that oxidative phosphorylation was the most significantly downregulated signaling pathway among insects with *PjDUF1* knocked down. *PjDUF1* expression impacted the activities of genes associated with oxidative phosphorylation. qRT‐PCR experiments and ATP level determination confirmed that the inhibition of *PjDUF1* expression affected oxidative phosphorylation rates. Oxidative phosphorylation is an important pathway for bioenergy production.^[^ ^61^ ^]^ Therefore, *PjDUF1* knockdown causes the insects to have insufficient energy to cope with insecticide stress. Subsequently, the RNAi experiments of two core genes (*SDHC* and *NDUFS3*) of oxidative phosphorylation confirmed that the level of oxidative phosphorylation can directly influence insect insecticide resistance. This is consistent with previous studies in other species demonstrating the role of oxidative phosphorylation in insecticide resistance.^[^ ^62^, ^63^, ^64^ ^]^ For example, fruit flies minimize damage induced by the neonicotinoid insecticide thiamethoxam by increasing oxidative phosphorylation levels.^[^ ^62^ ^]^ Furthermore, in *Drosophila*, enhanced oxidative phosphorylation capacity via successful expression of the yeast single‐subunit NADH dehydrogenase NDI1 significantly increases toxin resistance.^[^ ^65^ ^]^ While *PjDUF1* regulates insecticide resistance in ladybirds by mediating oxidative phosphorylation levels, the specific regulatory mechanism is unclear.

The GST Pull‐down/MS, CoIP/MS, Y2H, Luciferase Complementation and CoIP assays show that PjDUF1 regulates oxidative phosphorylation levels through interaction with cytochrome b‐c1 complex subunit Rieske, UQCRFS1. Oxidative phosphorylation is the process by which electrons are transferred from NADH or FADH2 to O2 through a series of electron carriers to create ATP. NADH dehydrogenase, also known as oxidative phosphorylation complex I, primarily functions in intermediary metabolism and regulates ATP synthesis.^[^ ^66^ ^]^ Mitochondrial complex II (succinate dehydrogenase) is crucial in regulating energy production; SDHC functions as an anchored subunit in the inner mitochondrial membrane.^[^ ^67^ ^]^ Complex III (cytochrome b‐c1 complex) catalyzes the oxidation of ubiquinol to ubiquinone, transferring electrons to cytochrome c and establishing a proton motive force used to synthesize adenosine triphosphate (ATP) using the F1Fo ATP synthase.^[^ ^68^ ^]^ *PjDUF1* regulates oxidative phosphorylation levels by influencing the oxidation of ubiquinol to ubiquinone and transferring electrons to cytochrome c. When normal oxidative phosphorylation is perturbed, cell homeostasis and energy formation are disrupted.^[^ ^69^ ^]^ When cell homeostasis and energy formation are altered, the individual becomes less resistant to environmental stresses like pesticides.^[^ ^70^ ^]^ Our multi‐platform validation robustly confirms the PjDUF1‐UQCRFS1 interaction. While these approaches demonstrate the functional significance of this binding, future site‐directed mutagenesis studies could precisely map the interaction interface and quantify residue‐specific contributions. Such work would further elucidate the structural basis for pesticide tolerance mediated by this interaction.

Previous studies have shown that the *PjDUF1* gene regulates *P. japonica* energy synthesis by binding to UQCRFS1. Therefore, in order to further verify the impact of *PjDUF1* gene silencing on energy synthesis, we evaluated the athletic and predatory abilities of the insects. Gene silencing showed that the knockdown of the *PjDUF1* gene significantly reduced the athletic and predatory abilities of ladybirds. This proves that *PjDUF1* regulated ladybird physiological characteristics by mediating oxidative phosphorylation. In addition, we identified mutual regulation between *PjDUF1* and *Acinetobacter* (Figure 8). The expression of *PjDUF1* was strongly correlated with the abundance of *Acinetobacter*. These results indicated that *Acinetobacter* can affect host physiological features by regulating the expression of the *PjDUF1* gene. Such coordination allows hosts to maintain symbioses even under external pressures, including insecticide exposure. Mediation of host insecticide resistance by symbiotic bacteria benefits the host insect and creates a more suitable environment for bacterial colonization. While our findings establish that the horizontally acquired *PjDUF1* gene significantly contributes to pesticide tolerance through modulation of oxidative phosphorylation pathways, several important questions remain open for future investigation. First, the observed correlation between *A. calcoaceticus* colonization and *PjDUF1* expression levels suggests potential bacterial‐host interactions that warrant deeper exploration. However, it should be emphasized that these data do not necessarily demonstrate direct bacterial manipulation of host gene expression – alternative explanations including metabolic cross‐talk or stress responses remain equally plausible. Second, the precise molecular mechanisms by which environmental bacteria might influence horizontally transferred gene expression in host organisms represent an exciting frontier for future research. Systematic identification of bacterial effectors, coupled with targeted host genetic screens, will be particularly valuable to dissect these complex interactions. Our current work provides a solid foundation for these subsequent mechanistic studies by clearly establishing the functional significance of this HGT event.

In conclusion, our study reveals that host insects have evolved pesticide resistance through synergistic interactions between horizontally acquired genetic elements and microbial symbionts. Specifically, coordinated regulation was identified between the horizontally transferred *PjDUF1* gene and *Acinetobacter* symbionts mediating dinotefuran tolerance. These findings offer new insights into co‐evolutionary adaptations where horizontal gene transfer (HGT) and symbiont partnerships jointly enable environmental stress responses. In contrast, our study also shows that *Acinetobacter* can be developed and used as a microbial agent to enhance the environmental fitness and predatory ability of predators with the *PjDUF1* gene.

## 3 Experimental Section

### Insects and Bacteria

*P. japonica* specimens were reared in mesh cages at 25 ± 1 °C with 60 ± 2% relative humidity (RH) under a long‐day regimen (14/10 h light/dark photoperiod). They were fed aphids (*Megoura crassicauda*). Sterile ladybirds were produced as previously described.^[^ ^30^, ^31^ ^]^ Specifically, the insects were fed an antibiotic cocktail of 500 µg/mL ampicillin (Putney), 50 µg mL^−1^ tetracycline (Sigma–Aldrich, St. Louis, MO, USA), and 200 µg mL^−1^ rifamycin (Sigma–Aldrich).

A strain of *Acinetobacter calcoaceticus* was isolated from the *P. japonica* gut (a ladybirds gut‐derived isolate from a traditionally reared population) by chromogenic medium (CHROMagar, France) and then cultured in lysogeny broth (LB) at 37°C, respectively. Using an inoculation loop, select the dominant single colonies that have developed on the petri dish. Inoculate them onto a fresh LB medium. Subsequently, perform further purification of the grown colonies to obtain a single strain. The V1–V9 hypervariable region of the 16S rRNA gene was amplified using the standard 27F (5’‐AGAGTTTGATCCTGGCTCAG‐3’) and 1492R (5’‐GGTTACCTTGTTACGACTT‐3’) primer pair for strain verification (Table S1, Supporting Information). Bacterial cells were harvested via centrifugation (3000 × *g* for 5 min) and then resuspended in sterile phosphate‐buffered saline (PBS) as previously described.^[^ ^30^ ^]^ The bacterial suspension was diluted to an optical density (OD)_600_ of 0.2–0.4, corresponding to ≈1 × 10^7^–2 × 10^7^ cells (Figure S2, Supporting Information). Sterilized food (1 mol L^−1^ Sucrose solution) was supplemented with the bacterial suspension to generate gnotobiotic insects. Mixed bacteria (MB) were harvested by grinding 30 conventionally reared adult *P. japonica* gut in 500 µL PBS.

### Insecticide Bioassays

Insecticide bioassays were performed using topical application and glass vial methods. *Topical application method*. One‐day‐old adult *P. japonica* individuals were treated with technical‐grade 96% dinotefuran (Macklin, Shanghai, China) as previously described.^[^ ^32^ ^]^ Briefly, a 1 µL droplet of insecticide solution (containing 1.83 ng dinotefuran) was applied to the abdominal region of each insect. There were 12 replicates per treatment, each containing eight to 11 insects. Insects were examined at 12, 24, 36, and 48 h to calculate the death rate. *Glass vial method*. Glass vials with an inner surface of 40.23 cm^2^ (4.5 cm height × 2.5 cm diameter) were evenly coated with 300 µL of 100 ng µL^−1^ dinotefuran solution in acetone. The same volume of acetone alone was used as a control. Each glass vial contained one insect, and eight to eleven replicate glass vials were used per treatment. Insects were examined at 12, 24, 36, and 48 h to calculate the death rate.

### Identification of HGTs

To identify insect genes that may have been horizontally acquired from bacteria, we employed a three‐step workflow following previously reported.^[^ ^33^ ^]^ Briefly, the first step (blast‐I) was the identification of highly homologous sequences of microbes (Data downloaded on April 7, 2022) in ladybirds by similarity search (E≤1E‐40 and identity value≥30%). In the second step, we constructed five outgroup taxa: plants (Data downloaded on April 25, 2022), fungi (Data downloaded on March 25, 2022), protozoa (Data downloaded on March 9, 2022), non‐insect arthropods (Data downloaded on June 25, 2022), and non‐arthropod metazoan (Data downloaded on June 25, 2022). The proteome used to build the outgroup taxa was obtained from the NCBI database (https://www.ncbi.nlm.nih.gov/public/) using Aspera software. Using the obtained bacteria‐insect high homology sequences as query sequences, the scores of bacteria‐insect similar sequences in other taxa were evaluated using the BLASTP program with the same threshold as blast‐I. If more than two of the five taxa had higher scores and identity values than bacteria, this gene was not a bacterial‐origin gene. The third step (blast‐III) was to compare candidate genes to the NCBI non‐redundant protein sequence database and select similar sequences (top 100) to construct an evolutionary tree. If a “gene tree” is inconsistent with a “species tree” it reflected phylogenetic relationships of related species, and a horizontal gene transfer event occurred.

### Gene Identification and Cloning

*PjDUF1* was identified in the recently sequenced *P. japonica* genome (https://www.ncbi.nlm.nih.gov/genome/?term=Propylea%20japonica) and annotated as a “putative uncharacterized protein”.^[^ ^34^ ^]^ The uncharacterized protein's predicted coding sequence (CDS) was manually corrected using previously published *P. japonica* transcriptomic data.^[^ ^35^ ^]^ cDNA fragments were generated with gene‐specific primers (Table S1, Supporting Information). Fragment sequences and completeness were verified via Sanger sequencing. The distribution of the DUF1963 functional domain was determined with InterPro (http://www.ebi.ac.uk/interpro/).^[^ ^36^ ^]^ The SMART online database (http://smart.embl.de/) predicted conserved domains encoded by *PjDUF1*. The 3D homology model of PjDUF1 was constructed using AlphaFold3.^[^ ^37^ ^]^

### Phylogenetic Analysis

The *PjDUF1* amino acid sequence was used as the query in BLASTP searches against the *P. japonica* genome at a threshold of E‐value < 10^−5^. *PjDUF1* amino acid sequence were subsequently used in a BLASTp search against the NCBI non‐redundant (NR) protein database (https://www.ncbi.nlm.nih.gov/, last accessed 7/7/2022). The top 100 most similar protein sequences were selected for phylogenetic analysis. The phylogenetic tree was constructed in MEGA6.0 using the maximum‐likelihood (ML) method with the (LG+G+I+F) model and 1000 bootstrap replicates. All positions containing gaps and missing data were eliminated. Trees were visualized with FigTree v1.4.4, iTOL (https://itol.embl.de/), and Adobe Photoshop CC software. To identify the distribution and evolutionary relationships of *PjDUF1* gene paralogs across Coleoptera species, blastp were used to search for *PjDUF1* homologs in 53 coleopteran species genome (Table S2, Supporting Information). In addition, to further determine the evolutionary relationship of the *PjDUF1* gene in the Coleoptera, a phylogenetic tree was constructed based on 53 species of Coleoptera and one outgroup species. To ensure the accuracy and completeness of the alignment results, the genomic sequences were first aligned using the multi‐sequence alignment tool MAFFT (https://mafft.cbrc.jp/alignment/software/). A global alignment strategy was adopted to obtain high‐quality alignment results. Subsequently, the RAxML software was used to construct the phylogenetic tree among the species. RAxML inferred the optimal evolutionary tree among the species using the maximum likelihood method (ML) and evaluated the stability and reliability of the tree through 1000 Bootstrap resampling iterations. In addition, the time of HGT events was further estimated using the life evolution time provided by Timetree 5 (https://timetree.org/).

### RNA Interference (RNAi)

Primer pairs specific for *PjDUF1* and *enhanced green fluorescent protein* (*eGFP*) were designed, each containing a T7 promoter at the 5′end (Table S1, Supporting Information). Double‐stranded RNAs (dsRNAs) targeting *PjDUF1* were synthesized in vitro from DNA templates generated via PCR using the T7 RiboMAX Express RNAi System (Promega, Madison, WI, USA). RNAi was conducted as previously described.^[^ ^38^ ^]^ Briefly, 1‐day‐old *P. japonica* adults were injected with 1.5–2 µg of dsRNA in the abdominal hemocoel. Control insects were injected with the same dose of *eGFP*‐dsRNA. The interference efficiency was calculated 2 d later with quantitative reverse‐transcription (qRT)‐PCR as described below.

### Transcriptomic Analysis of *PjDUF1* Knockdowns

Insects confirmed to have *PjDUF1* silenced after treatment with PjDUF1‐RNAi were analyzed with transcriptomic analysis. Total RNA was extracted as described above (RNA extraction and qRT‐PCR), and RNA libraries were constructed with the Truseq RNA sample prep kit (Illumina, San Diego, CA, USA). Sequencing was conducted on the Illumina NovaSeq 6000 platform. There were three biological replicates of *PjDUF1*‐RNAi insects and the *eGFP*‐RNAi control. Clean reads were mapped to the *P. japonica* genome using TopHat2 (http://tophat.cbcb.umd.edu/). Cufflinks (http://cole‐trapnelllab.github.io/cufflinks/) was then used to assemble and splice the mapped reads. Functional gene/transcript annotation was performed using the NCBI NR (https://www.ncbi.nlm.nih.gov/public/), Swiss‐Prot (ftp://ftp.uniprot.org/pub/databases/uniprot/current_release/knowledgebase/complete/uniprot_sprot.fasta.gz), Pfam (http://pfam.xfam.org/), EggNOG (http://eggnogdb.embl.de/#/app/home), Gene Ontology (GO) (http://www.geneontology.org/), and Kyoto Encyclopedia of Genes and Genomes (KEGG) (http://www.genome.jp/kegg/) databases. Differentially expressed genes (DEGs) were called with the ‘DESeq2’ R package (http://bioconductor.org/packages/stats/bioc/DESeq2/) at fold change (FC) ≥2 and false‐discovery rate (FDR) < 0.05 following the standard workflow.

### RNA Extraction and qRT‐PCR

Total RNA was extracted using TRIzol reagent (Invitrogen, Waltham, MA, USA) following previously published methods.^[^ ^35^ ^]^ Each sample was extracted in 1 mL TRIzol reagent. RNA quantity and quality were measured with a Nanodrop 2000 (Thermo Fisher Scientific, Waltham, MA, USA) and 1.5% agarose gel electrophoresis. For each sample, 1 µg of RNA was used as the cDNA template using the PrimeScript™ RT reagent Kit with gDNA Eraser (Takara, DaLian, China). Target gene expression levels were quantified on a LightCycler 480 (Roche, Basel, Switzerland). Relative gene expression was calculated with the 2^−ΔΔCt^ method using *RPS18* as the internal control.^[^ ^39^, ^40^ ^]^ All primers for qRT‐PCR are shown in Table S1 (Supporting Information).

### Bacterial Load Quantification

Total genomic DNA (gDNA) was extracted from whole adult *P. japonica* individuals using the FastDNA SPIN Kit (MP Biomedicals, Santa Ana, CA, USA) as previously described.^[^ ^3^ ^]^ Quantitative PCR (qPCR) was performed on a StepOnePlus™ Real‐Time PCR System (Applied Biosystems, Foster City, CA, USA) to determine the relative total and individual bacterial load using the primers shown in Table S1 (Supporting Information).

### High‐Throughput 16S rRNA Sequencing

DNA was extracted from 8 samples, each containing 12 *P. japonica* adults. Insects were surface‐sterilized with 75% ethanol for 2 min, then rinsed three times with sterilized deionized water. Samples were homogenized in a sterile grinding pestle and frozen at −80°C before DNA extraction. Total DNA was extracted as described above (gDNA isolation and genome fragment cloning). The V3–V4 hypervariable region of the 16S rRNA gene was amplified using the standard 338F/806R primer pair (Table S1, Supporting Information). PCR amplification, fluorescent quantitation, Illumina library construction, and Illumina sequencing were performed following the standard protocols of Majorbio Bio‐Pharm Technology Co. Ltd. Sequencing data were processed with DADA2/Deblur to obtain amplicon sequence variants (ASVs). Hierarchical clustering, community diversity, community composition, and species difference analyses were performed on the Majorbio Cloud Platform (https://cloud.majorbio.com/page/tools.html).

### Fluorescence In Situ Hybridization (FISH)

*Acinetobacter* localization was examined with FISH in *P. japonica* at the egg, second instar larva, fourth instar larva, pupa, and adult stages as previously described.^[^ ^41^ ^]^ Each sample was embedded in paraffin and cut into slices of 5 µm in thickness with an RM 2016 microtome (Leica Microsystems, Wetzlar, Germany). The resulting samples were digested, decolorized, and hybridized overnight. *Acinetobacter* symbionts were detected with the Cy3‐labeled Burk16S probe (5′‐CGGTATTCCTCCAGATCTCTACGCATTTCACCGCTACACCTGGAA‐3′). Samples were counterstained with 4', 6‐diamidino‐2‐phenylindole (DAPI) to label the insect cell nuclei. Images were acquired on an Eclipse Ci microscope (Nikon, Tokyo, Japan).

### gDNA Isolation and Genome Fragment Cloning

gDNA was extracted from *P. japonica* adults using the TIANamp Genomic DNA Extraction Kit DP304 following the manufacturer's instructions (TIANGEN, Beijing, China). Primers were designed with Primer Premier 6.0 to target the regions between *PjDUF1* and the upstream and downstream neighboring genes (Table S1, Supporting Information); PCR was conducted to amplify the fragments. The correctness and completeness of the fragments were verified with Sanger sequencing. Amplified sequences were compared with the *P. japonica* genomic sequence using DNAMAN software.

### Heterologous Expression

The recombinant plasmid PjDUF1‐Flag‐pFastBac1 was expressed in *Spodoptera frugiperda* Sf9 cells using a Bac‐to‐Bac Baculovirus Expression System. The CDS of *PjDUF1* was synthesized, and inserted into the pFastBac1 vector, and the target gene was inserted downstream into the Flag fusion expression to produce the recombinant plasmid. The insertion sequence was validated by Sanger sequencing. The recombinant plasmid of PjDUF1‐Flag‐pFastBac1 was transformed into *Escherichia coli* (DH10Bac). Recombinant Bacmid DNA was extracted using Beyotime Baculovirus Shuttle Vector Bacmid Mini Preparation Kit (Beyotime Biotechnology, China). The extracted recombinant Bacmid was transfected into *Spodoptera frugiperda* Sf9 cells using LipoInsect™ Transfection Reagent (Beyotime Biotechnology, China). Seventy‐two hours after transfection, protein samples were extracted from the harvested cells. The protein concentrations were measured using a BCA assay.

### Metabolic Experiments

The assay of the initial PjDUF1 activity survey was based on the methods described previously with modification.^[^ ^13^ ^]^ The metabolic activities of PjDUF1 was assayed by incubation of Sf9‐expressed purified PjDUF1 protein (5 µg) in 250 µl Tris‐HCl buffer (0.2 m, pH 7.5) containing an NADPH‐regeneration system (Promega) and 0.2 mm of a dinotefuran at 30°C for 1 h and each reaction was replicated twelve times. The reaction mixture without the PjDUF1 protein was used as the control. The reactions were stopped by adding 250 µL of methanol (HPLC grade, Thermo Fisher Scientific) and were centrifuged at 12 000 g for 10 min. Then, the supernatant was analyzed by Shimadzu High Performance Liquid Chromatography with LC‐20AD pump system, equipped with RF‐20A fluorescence detector. The column for the analysis of dinotefuran in the samples was Shim‐pack GIST‐HP C18 (3 µm, 2.1 mm × 75 mm, Shimadzu, Japan). The mobile phase consisted of methanol and ultra‐pure water (v/v) = 60:40, with a flow rate of 0.25 ml min^−1^, an injection volume of 10 µL and Column temperature 40°C.

### Determination of Oxidative Phosphorylation Level

The oxidative phosphorylation levels were estimated by measuring energy production. The ATP, ADP, and AMP levels were determined by Ultra Performance Liquid Chromatography/Mass Spectrometry (UPLC‐MS/MS). The insect sample was weighed into a 2 mL brown centrifuge tube, and then 0.5 mL of pre‐cooled 1% perchloric acid aqueous solution was added. Vortex oscillation occurred at 4°C for 1 min, followed by low‐temperature ultrasonic extraction for 30 min at 100 Hz. After completion, samples were centrifuged at 4°C at 13000 r/min for 15 min. Then, an appropriate amount of supernatant was taken through a 0.22 µm filter membrane, diluted with distilled water two times, and mixed into a brown sample bottle for characterization. The chromatographic column was performed on a Waters HSS T3 (2.1 mm × 100 mm, 1.8 µm), column at 30°C. The mobile phase was 1 mmol ammonium acetate aqueous solution (pH = 10) and acetonitrile. The flow rate was 1.0 mL min^−1^. The UV detection wavelength was 254 nm.

### Oxidative Phosphorylation Inhibitor

In order to verify the impact of reduced oxidative phosphorylation levels on the tolerance of insects to insecticides tolerance of ladybirds, the inhibitor of the mitochondrial complex III (antimycin A: purchased from Pyeast Bio. Co., Ltd.) was selected to inhibit the oxidative phosphorylation level of the ladybug. Antimycin A is diluted with dimethyl Sulfoxide (DMSO). According to the previous reports,^[^ ^42^ ^]^ we used 1 µl (10 ng µl^−1^, 5 ng µl^−1^, 2.5 ng µl^−1^ and 1.25 ng µl^−1^) antimycin A solution to treat the ladybirds by *Topical application method*. After 24 h of treatment, a 1 µL droplet of dinotefuran solution (containing 1.83 ng dinotefuran) was applied to the abdominal region of each insect. After 24 h of DMSO treatment, 1.83 ng dinotefuran treatment served as the control group.

### Antibody Preparation and Western Blot

Custom‐made polyclonal antibodies against PjDUF1 (predicted size, 28.2 kDa) proteins were produced by Wuhan GeneCreate Biological Engineering Co., Ltd., following previously described methods.^[^ ^43^ ^]^ Rabbits were immunized four times using the purified recombinant protein. The antiserum from the last bleed was affinity purified and tested by enzyme‐linked immunosorbent assay. The specificity of polyclonal antibodies was confirmed by western blot. Briefly, proteins separated by 10% SDS‐PAGE were transferred to 0.22 µm PVDF membranes using a Trans‐Blot SD Semi‐Dry Electrophoretic Transfer Cell (Bio‐Rad, USA). The PVDF membranes were incubated with primary antibodies at 4°C overnight. The PVDF membrane was fully washed by TBST four times, 10 min per time. Then, the PVDF membrane was immersed in the secondary antibody incubation solution and incubated in a shaking table at 37°C for 2 h. The ECL immunoblotting detection reagent (Sangon Biotech, Shanghai, China) was used to visualize enhanced chemiluminescence color bands. The signal was detected using a Tanon‐5200 Chemiluminescent Imaging System (Tanon, Shanghai, China).

### Homology Modelling and Docking

The 3D structure of the PjDUF1 and UQCRFS1 protein was constructed by AlphaFold 3 (https://golgi.sandbox.google.com/).^[^ ^44^ ^]^ The complex structure of PjDUF1 with UQCRFS1 was generated using the protein–protein docking tool HADDOCK software.^[^ ^45^ ^]^ The amino acids on the protein surface were set as interacting amino acids, and the reliability of the docking conformation was determined by statistical scoring. Ten clusters of docked positions of PjDUF1 and UQCRFS1 were determined using HADDOCK. Among them, based on the HADDOCK score and Z‐value, the best structure was downloaded from the PDB. Finally, the docked conformations were visualized and analyzed using ChimeraX 1.10.^[^ ^46^ ^]^

### GST Pull‐Down Assays

The GST‐tagged fusion protein GST‐PjDUF1 was used as a protein bait (constructed and synthesized by Henan Writegene Biotechnology Co., Ltd.). Gel staining was conducted with Coomassie Blue (eStainTM L1 Protein Staining System, GenScript) to characterize the GST‐PjDUF1 fusion protein. Purified GST was a negative control. Prey proteins were obtained by immobilizing and binding bait proteins to immobilize bait proteins using magnetic beads. Protein interactions with the bait protein were identified by mass spectrometry.

### Co‐Immunoprecipitation (CO‐IP)

The PjDUF1‐Flag‐pFastBac1 fusion protein was constructed as a protein bait (constructed and synthesized by Henan Writegene Biotechnology Co., LTD). The bait protein was expressed in *Spodoptera frugiperda* Sf9 cells (Invitrogen). Prey Proteins were obtained by BeyoMag™ Protein A+G magnetic beads and the FLAG antibody (Rabbit) diluted with PBS. Specifically, BeyoMag™ Protein A+G magnetic beads were supplemented with 4 µg FLAG‐Tag Polyclonal Antibody (Rabbit) diluted with PBS. The mixture was incubated in a rotating mixer at room temperature for one hour, separated on a magnetic rack for 10 s after incubation, and the supernatant was discarded. A total of 100 µL of protein lysate was used as the Input control, and the remaining 900 µL was added into the FLAG antiboy‐magnetic bead complex, mixed, and placed on a rotating mixing apparatus and incubated at room temperature for two hours. Following incubation, the magnetic beads were separated on a magnetic rack for 10 s, and the supernatant was discarded. A total of 1 mL of immunoprecipitation washing buffer was added into the magnetic bead complex, washed for 2 min, and repeated washing four times. 80 µL PBS and 5× protein loading buffer were added to the magnetic beads, boiled for 10 min, and the supernatant was separated on the magnetic rack for mass spectrometry.

### Yeast Two‐Hybrid Assays

Based on the genome sequences of *P. japonica*, a pair of primers, including restriction enzyme sites (Table S1, Supporting Information), were designed to insert the full‐length coding sequences (CDSs) of the bait protein and prey protein. PCR products were confirmed by Sanger sequencing. The target band was purified using a Wizard® SV Gel and PCR Clean‐Up System (Promega, Madison, USA). The validated target fragments were independently cloned into the pGADT7 and pGBKT7 vectors (Coolaber, Beijing, China) using suitable restriction sites (*Nde*I and *Sma*I). All recombinant plasmids were transferred to the Y2HGold yeast strain. Clones with the desired DNA fragment were screened for auto‐activation on SD medium, and protein interactions were detected on SD medium lacking Ade, His, Leu, and Trp. The Y2HGold (pGBKT7‐53+ pGADT7‐T) yeast strain was used as a positive control. The Y2HGold (pGBKT7‐Lam+ pGADT7‐T) yeast strain was a negative control. The X‐α‐Gal medium was used to detect colonies with positive hybridization.

### Dual‐Luciferase Assay

The CDSs of two target proteins were cloned into p1300‐nLuc and p1300‐cLuc vectors. The constructed two experimental plasmids, two no‐load plasmids (p1300‐nLuc, p1300‐cLuc), and two positive control plasmids (P1300‐nLU‐C T, P1300‐CLUC‐p53) were transformed into *Agrobacterium* strain GV3101 (pSoup) and then co‐transfected into *Nicotiana benthamiana* leaves. After 36–48 h of infiltration, tobacco leaves were injected with 1 mmol/LD‐luciferin potassium substrate to characterize luciferase activity by Tanon‐5200 Chemiluminescent Imaging System (Tanon).

### The Assessment of Athletic Ability and Feeding Amount of *P. Japonica*. 

We placed the Ladybirds into a 15‐cm Petri dish, and recorded their movement trajectory for 12 h. To measure the Athletic ability, we collected 12 insect movement information for each treatment. According to previous reports,^[^ ^47^ ^]^ the position information of ladybirds was extracted and analyzed by Deeplabcut software (v2.2.1). We first used Deeplabcut software to train, recognize, and calculate insect behavior. We isolated 500 frames of photos from each video (using 10 videos), manually labeled the location of the insects in each frame, and conducted simulation training. The model was trained on 3 00 000 training iterations. The test data was predicted and validated, and the performance of the trained model was evaluated. Then, each video was analyzed, and the insect coordinates of each frame of each video were obtained. After distance conversion, the trajectory diagram exhibited the average speed, moving distance, moving time, and rest time of the insect. The trajectory diagram was converted to a trajectory heat map using Matlab code (https://www.mathworks.com/discovery/matlab‐code.html). We selected 45 adult ladybirds for each treatment and placed them in 15‐cm Petri dishes for individual feeding. A total of 200 adult aphids, *Megoura crassicauda*, were added, and the number of feeding aphids was counted every two days over nine consecutive days.

## Conflict of Interest

The authors declare no conflict of interest.

## Author Contributions

N.H.F., X.G., J.L., and J.C. designed the experiments. N.H.F. performed the experiments. N.H.F. and X.G. analyzed the experiments. Z.Z., Z.T., L.W., K.Z., D.L., and J.J. assisted in experiments. N.H.F. wrote the manuscript. X.G., J.L., and J.C. supervised the work. All authors have read and agreed to the published version of the manuscript.

## Supporting information



Supporting Information

Supplemental Table 1

## Data Availability

All raw High‐throughput Transcriptome (accession no. PRJNA1049653) and 16S rRNA sequencing data (accession no. PRJNA1049732) are uploaded to the NCBI database and available upon publication.

## References

[1] M. Groussin , F. Mazel , E. J. Alm , Cell Host Microbe 2020, 28, 12. 32645351 10.1016/j.chom.2020.06.013

[2] C. Dale , N. A. Moran , Cell 2006, 126, 453. 16901780 10.1016/j.cell.2006.07.014

[3] T. Cai , P. Nadal‐Jimenez , Y. Gao , H. Arai , C. Li , C. Su , K. C. King , S. He , J. Li , G. D. D. Hurst , H. Wan , ISME J. 2024, 18, wrae194. 39375012 10.1093/ismejo/wrae194 PMC11491930

[4] Y. Sato , S. Jang , K. Takeshita , H. Itoh , H. Koike , K. Tago , M. Hayatsu , T. Hori , Y. Kikuchi , Nat. Commun. 2021, 12, 6432. 34741016 10.1038/s41467-021-26649-2 PMC8571283

[5] J. Hemingway , L. Field , J. Vontas , Science 2002, 298, 96. 12364782 10.1126/science.1078052

[6] A. Alyokhin , Y. H. Chen , Curr. Opin. Insect Sci. 2017, 21, 33. 28822486 10.1016/j.cois.2017.04.006

[7] E. Rosenberg , I. Zilber‐Rosenberg , Microbiome 2018, 6, 78. 29695294 10.1186/s40168-018-0457-9 PMC5922317

[8] P. J. Keeling , J. D. Palmer , Nat. Rev. Genet. 2008, 9, 605. 18591983 10.1038/nrg2386

[9] S. M. Soucy , J. Huang , J. P. Gogarten , Nat. Rev. Genet. 2015, 16, 472. 26184597 10.1038/nrg3962

[10] Y. Li , Z. Liu , C. Liu , Z. Shi , L. Pang , C. Chen , Y. Chen , R. Pan , W. Zhou , X.‐X. Chen , A. Rokas , J. Huang , X.‐X. Shen , Cell 2022, 185, P2975. 10.1016/j.cell.2022.06.014 PMC9357157 35853453

[11] J. C. Dunning Hotopp , M. E. Clark , D. C. Oliveira , J. M. Foster , P. Fischer , M. C. Muñoz Torres , J. D. Giebel , N. Kumar , N. Ishmael , S. Wang , J. Ingram , R. V. Nene , J. Shepard , J. Tomkins , S. Richards , D. J. Spiro , E. Ghedin , B. E. Slatko , H. Tettelin , J. H. Werren , Science 2007, 317, 1753. 17761848 10.1126/science.1142490

[12] X. Dai , T. Kiuchi , Y. Zhou , S. Jia , Y. Xu , S. Katsuma , T. Shimada , H. Wang , Mol. Biol. Evol. 2021, 38, 2897. 33739418 10.1093/molbev/msab080 PMC8233494

[13] J. Xia , Z. Guo , Z. Yang , H. Han , S. Wang , H. Xu , X. Yang , F. Yang , Q. Wu , W. Xie , X. Zhou , W. Dermauw , T. C. J. Turlings , Y. Zhang , Cell 2021, 184, 1693. 33770502 10.1016/j.cell.2021.02.014

[14] Y. He , B. Zhang , Y. Wu , J. Ouyang , M. Huang , S. Lu , H. Sun , T. Zhang , Environ. Pollut. 2021, 291, 118117. 34534832 10.1016/j.envpol.2021.118117

[15] Y. Zhou , X. Lu , B. Yu , D. Wang , C. Zhao , Q. Yang , Q. Zhang , Y. Tan , X. Wang , J. Guo , Sci. Total Environ. 2021, 782, 146803. 33848872 10.1016/j.scitotenv.2021.146803

[16] Q. Zhang , Z. Lu , C. H. Chang , C. Yu , X. Wang , C. Lu , Environ. Int. 2019, 126, 672. 30856454 10.1016/j.envint.2019.02.051

[17] M. Xu , Z. Zhang , Z. Li , S. Kan , Z. Liu , D. Wang , Q. Liu , H. Zhang , Sci. Total Environ. 2021, 773, 145582. 33582343 10.1016/j.scitotenv.2021.145582

[18] K. Matsuda , M. Ihara , D. B. Sattelle , Annu Rev. Pharmacol. Toxicol. 2020, 60, 241. 31914891 10.1146/annurev-pharmtox-010818-021747

[19] L. D. Tang , B. L. Qiu , A. G. Cuthbertson , S. X. Ren , Pestic. Biochem. Physiol. 2015, 123, 87. 26267056 10.1016/j.pestbp.2015.03.008

[20] L. D. Tang , X. M. Wang , F. L. Jin , B. L. Qiu , J. H. Wu , S. X. Ren , PLoS One 2014, 9, 100946. 10.1371/journal.pone.0100946 PMC4069172 24959827

[21] N. Lv , R. Li , S. Cheng , L. Zhang , P. Liang , X. Gao , BMC Biol. 2023, 21, 86. 37069589 10.1186/s12915-023-01586-2 PMC10111731

[22] H. Wang , C. Zhang , P. Cheng , Y. Wang , H. Liu , H. Wang , H. Wang , M. Gong , Microbiologyopen 2021, 10, 1177. 10.1002/mbo3.1177 PMC8087943 33970535

[23] X. Gao , F. Hu , S. Zhang , J. Luo , X. Zhu , L. Wang , K. Zhang , D. Li , J. Ji , L. Niu , C. Wu , J. Cui , Sci. Total Environ. 2021, 790, 147847. 34082325 10.1016/j.scitotenv.2021.147847

[24] C.‐R. Lee , J. H. Lee , M. Park , K. S. Park , I. K Bae , Y. B. Kim , C.‐J. Cha , B. C. Jeong , S. H. Lee , Front. Cell Infect. Microbiol. 2017, 7, 55. 28348979 10.3389/fcimb.2017.00055 PMC5346588

[25] H. C. Flemming , J. Wingender , U. Szewzyk , P. Steinberg , S. A. Rice , S. Kjelleberg , Nat. Rev. Microbiol. 2016, 14, 563. 27510863 10.1038/nrmicro.2016.94

[26] A. L. Sezmis , L. C. Woods , A. Y. Peleg , M. J. McDonald , Mol. Biol. Evol. 2023, 40, msad028. 36788632 10.1093/molbev/msad028 PMC9985319

[27] M. Hamidian , D. P. Hancock , R. M. Hall , J. Antimicrob. Chemother. 2013, 68, 244. 22915466 10.1093/jac/dks345

[28] V. Riva , G. Patania , F. Riva , L. Vergani , E. Crotti , F. Mapelli , Antibiotics 2022, 11, 1231. 36140010 10.3390/antibiotics11091231 PMC9495178

[29] M. Maeusli , B. Lee , S. Miller , Z. Reyna , P. Lu , J. Yan , A. Ulhaq , N. Skandalis , B. Spellberg , B. Luna , mSphere. 2020, 5, 00329. 10.1128/mSphere.00329-20 PMC7253597 32461272

[30] Y. Jia , S. Jin , K. Hu , L. Geng , C. Han , R. Kang , Y. Pang , E. Ling , E. K. Tan , Y. Pan , W. Liu , Nat. Commun. 2021, 12, 2698. 33976215 10.1038/s41467-021-23041-y PMC8113466

[31] C. E. Schretter , J. Vielmetter , I. Bartos , Z. Marka , S. Marka , S. Argade , S. K. Mazmanian , Nature 2018, 563, 402. 30356215 10.1038/s41586-018-0634-9 PMC6237646

[32] Y. Zhang , T. Cai , Z. Ren , Y. Liu , M. Yuan , Y. Cai , C. Yu , R. Shu , S. He , J. Li , A. C. N. Wong , H. Wan , Isme J. 2021, 15, 3693. 34188180 10.1038/s41396-021-01046-1 PMC8630103

[33] Z. W. Li , Y. H. Shen , Z. H. Xiang , Z. Zhang , BMC Evol. Biol. 2011, 11, 356. 22151541 10.1186/1471-2148-11-356 PMC3252269

[34] L. Zhang , S. Li , J. Luo , P. Du , L. Wu , Y. Li , X. Zhu , L. Wang , S. Zhang , J. Cui , Mol. Ecol. Resour. 2020, 20, 292. 31599108 10.1111/1755-0998.13100

[35] N. HuangFu , X. Zhu , G. Chang , L. Wang , D. Li , K. Zhang , X. Gao , J. Ji , J. Luo , J. Cui , Genomics. 2021, 113, 2877. 34116170 10.1016/j.ygeno.2021.06.002

[36] T. Paysan‐Lafosse , M. Blum , S. Chuguransky , T. Grego , B. L. Pinto , G. A. Salazar , M. L. Bileschi , P. Bork , A. Bridge , L. Colwell , J. Gough , D. H. Haft , I. Letunic , A. Marchler‐Bauer , H. Mi , D. A. Natale , C. A. Orengo , A. P. Pandurangan , C. Rivoire , C. J. A. Sigrist , I. Sillitoe , N. Thanki , P. D. Thomas , S. C. E. Tosatto , C. H. Wu , A. Bateman , Nucleic Acids Res. 2023, 51, D418. 36350672 10.1093/nar/gkac993 PMC9825450

[37] R. Roy , A.‐H. HM , Nat. Struct. Mol. Biol. 2024, 31, 997. 38977902 10.1038/s41594-024-01350-2

[38] N. Huangfu , X. Zhu , L. Wang , K. Zhang , D. Li , L. Chen , X. Gao , L. Niu , M. Gao , J. Ji , J. Luo , J. Cui , J. Agric. Food Chem. 2023, 71, 300. 36538395 10.1021/acs.jafc.2c07433

[39] K. J. Livak , T. D. Schmittgen , Methods 2001, 25, 402. 11846609 10.1006/meth.2001.1262

[40] J. Lü , S. Chen , M. Guo , C. Ye , B. Qiu , C. Yang , H. Pan , PLoS One 2018, 27, 0208027. 10.1371/journal.pone.0208027 PMC6258549 30481225

[41] B. Weiss , M. Kaltenpoth , Front. Microbiol. 2016, 7, 1486. 27713733 10.3389/fmicb.2016.01486 PMC5031591

[42] E. M. Choi , W. W. Jung , K. S. Suh , Mol. Med. Rep. 2015, 11, 746. 25334089 10.3892/mmr.2014.2721

[43] F.‐R. Ren , X. Sun , T.‐Y. Wang , Y.‐L. Yao , Y.‐Z. Huang , X. Zhang , J.‐B. Luan , ISME J. 2020, 14, 2542. 32572143 10.1038/s41396-020-0704-5 PMC7490365

[44] J. Abramson , J. Adler , J. Dunger , R. Evans , T. Green , A. Pritzel , O. Ronneberger , L. Willmore , A. J. Ballard , J. Bambrick , S. W. Bodenstein , D. A. Evans , C.‐C. Hung , M. O'Neill , D. Reiman , K. Tunyasuvunakool , Z. Wu , A. Zemgulyte , E. Arvaniti , C. Beattie , O. Bertolli , A. Bridgland , A. Cherepanov , M. Congreve , A. I. Cowen‐Rivers , A. Cowie , M. Figurnov , F. B. Fuchs , H. Gladman , R. Jain , et al., Nature 2024, 630, 493. 38718835 10.1038/s41586-024-07487-w PMC11168924

[45] R. V. Honorato , M. E. Trellet , B. Jiménez‐García , J. J. Schaarschmidt , M. Giulini , V. Reys , P. I. Koukos , J. P. G. L. M. Rodrigues , E. Karaca , G. C. P. van Zundert , J. Roel‐Touris , C. W. van Noort , Z. Jandová , A. S. J. Melquiond , A. M. J. J. Bonvin , Nat. Protoc. 2024, 19, 3219. 38886530 10.1038/s41596-024-01011-0

[46] T. D. Goddard , C. C. Huang , E. C. Meng , E. F. Pettersen , G. S. Couch , J. H. Morris , T. E. Ferrin , Protein Sci. 2018, 27, 14. 28710774 10.1002/pro.3235 PMC5734306

[47] J. Lauer , M. Zhou , S. Ye , W. Menegas , S. Schneider , T. Nath , M. M. Rahman , V. Di Santo , D. Soberanes , G. Feng , V. N. Murthy , G. Lauder , C. Dulac , M. W. Mathis , A. Mathis , Nat. Methods 2022, 19, 496. 35414125 10.1038/s41592-022-01443-0 PMC9007739

[48] C. Tang , X. Hu , J. Tang , L. wang , X. Liu , Y. Peng , Y. Xia , J. Xie , Commun. Biol. 2024, 7, 1184. 39300313 10.1038/s42003-024-06779-1 PMC11412983

[49] R. Palmen , K. J. Hellingwerf , Gene 1997, 192, 179. 9224889 10.1016/s0378-1119(97)00042-5

[50] T. Z. Jing , F. H. Qi , Z. Y. Wang , Microbiome 2020, 8, 38. 32178739 10.1186/s40168-020-00823-y PMC7077154

[51] J. H. Jeon , K. M. Jang , J. H. Lee , L. W. Kang , S. H. Lee , Sci. Total Environ. 2023, 857, 159497. 36257427 10.1016/j.scitotenv.2022.159497

[52] M. Touchon , J. Cury , E.‐J. Yoon , L. Krizova , G. C. Cerqueira , C. Murphy , M. Feldgarden , J. Wortman , D. Clermont , T. Lambert , C. Grillot‐Courvalin , A. Nemec , P. Courvalin , E. P. C. Rocha , Genome Biol. Evol. 2014, 6, 2866. 25313016 10.1093/gbe/evu225 PMC4224351

[53] S. R. Partridge , S. M. Kwong , N. Firth , S. O. Jensen , Clin. Microbiol. Rev. 2018, 31, 00088. 10.1128/CMR.00088-17 PMC6148190 30068738

[54] H.‐S. Li , X.‐F. Tang , Y.‐H Huang , Z.‐Y. Xu , M.‐L. Chen , X.‐Y. Du , B.‐Y. Qiu , P.‐T. Chen , W. Zhang , A. Slipinski , H. E. Escalona , R. M. Waterhouse , A. Zwick , H. Pang , BMC Biol. 2021, 19, 7. 33446206 10.1186/s12915-020-00945-7 PMC7807722

[55] E. Novakova , N. A. Moran , Mol. Biol. Evol. 2012, 29, 313. 21878683 10.1093/molbev/msr206

[56] A. Nakabachi , K. Ishida , Y. Hongoh , M. Ohkuma , S. Y. Miyagishima , Curr. Biol. 2014, 24, R640. 25050957 10.1016/j.cub.2014.06.038

[57] Z. Yang , Z. Guo , C. Gong , J. Xia , Y. Hu , J. Zhong , X. Yang , W. Xie , S. Wang , Q. Wu , W. Ye , B. Liu , X. Zhou , T. C. J. Turlings , Y. Zhang , Sci. Adv. 2024, 10, adi3105. 10.1126/sciadv.adi3105 PMC10836729 38306427

[58] C. Gong , Z. Guo , Y. Hu , Z. Yang , J. Xia , X. Yang , W. Xie , S. Wang , Q. Wu , W. Ye , X. Zhou , T. C. J. Turlings , Y. Zhang , Adv. Sci. 2024, 11, 2306653. 10.1002/advs.202306653 PMC10933598 38145364

[59] Y. Hu , C. Gong , Z. Yang , H. Han , T. Tian , X. Yang , W. Xie , S. Wang , Q. Wu , X. Zhou , T. C. J. Turlings , Z. Guo , Y. Zhang , Adv. Sci. 2025, 12, 2502193. 10.1002/advs.202502193 PMC12021119 40019366

[60] D. D. McKenna , S. Shin , D. Ahrens , M. Balke , C. Beza‐Beza , D. J. Clarke , A. Donath , H. E. Escalona , F. Friedrich , H. Letsch , S. Liu , D. Maddison , C. Mayer , B. Misof , P. J. Murin , O. Niehuis , R. S. Peters , L. Podsiadlowski , H. Pohl , E. D. Scully , E. V. Yan , X. Zhou , A. Slipinski , R. G. Beutel , Proc. Natl. Acad. Sci. U.S.A. 2019, 116, 24729. 31740605 10.1073/pnas.1909655116 PMC6900523

[61] A. L. Lehninger , C. L. Wadkins , C. Cooper , T. M. Devlin , G. J. L. Jr , Science 1958, 128, 450. 13568809 10.1126/science.128.3322.450

[62] X. Li , M. Li , X. Xue , X. Wang , Chemosphere 2023, 338, 139448. 37437626 10.1016/j.chemosphere.2023.139448

[63] C. Zhang , Q. Shi , T. Li , P. Cheng , X. Guo , X. Song , M. Gong , PLoS Negl. Trop. Dis. 2021, 15, 0009237. 10.1371/journal.pntd.0009237 PMC7993597 33764997

[64] L. Xu , X. Xu , H. Kuang , Y. Liu , C. Xu , X. Wu , Anal. Chem. 2023, 95, 3108. 36693709 10.1021/acs.analchem.2c05754

[65] A. Sanz , M. Soikkeli , M. Portero‐Otín , A. Wilson , E. Kemppainen , G. McIlroy , S. Ellilä , K. K. Kemppainen , T. Tuomela , M. Lakanmaa , E. Kiviranta , R. Stefanatos , E. Dufour , B. Hutz , A. Naudí , M. Jové , A. Zeb , S. Vartiainen , A. Matsuno‐Yagi , T. Yagi , P. Rustin , R. Pamplona , H. T. Jacobs , Proc Natl Acad Sci U.S.A. 2010, 107, 9105. 20435911 10.1073/pnas.0911539107 PMC2889079

[66] C. Perier , K. Tieu , C. Guégan , C. Caspersen , V. Jackson‐Lewis , V. Carelli , A. Martinuzzi , M. Hirano , S. Przedborski , M. Vila , Proc. Natl. Acad. Sci. U.S.A. 2005, 102, 19126. 16365298 10.1073/pnas.0508215102 PMC1323177

[67] J. L. Ding , X. H. Li , J. H. Lei , M. G. Feng , S. H. Ying , Microbiol. Spectr. 2022, 10, 289122. 10.1128/spectrum.02891-22 PMC9602434 35972281

[68] B. Wiseman , R. G. Nitharwal , O. Fedotovskaya , J. Schäfer , H. Guo , Q. Kuang , S. Benlekbir , D. Sjöstrand , P. Ädelroth , J. L. Rubinstein , P. Brzezinski , M. Högbom , Nat. Struct. Mol. Biol. 2018, 25, 1128. 30518849 10.1038/s41594-018-0160-3

[69] M. J. Dawkins , J. D. Judah , K. R. Rees , Nature 1958, 27, 875. 10.1038/182875b0 13590150

[70] H. Jiang , X. Meng , N. Zhang , H. Ge , J. Wei , K. Qian , Y. Zheng , Y. Park , S. Reddy Palli , J. Wang , Proc Natl Acad Sci U S A. 2023, 120, 2214038120. 10.1073/pnas.2214038120 PMC10013871 36853946

