# Supplementary material for: Gut Microbe‐Driven Resistance Mechanisms in Propylea Japonica: Insights from Horizontal Gene Transfer and Oxidative Phosphorylation
Source: Adv Sci (Weinh). 2025 Dec 12;13(11):e20326. doi: 10.1002/advs.202520326 (PMC12931235; doi:10.1002/advs.202520326)
Supplement: Supplementary file 1 — Supporting Information [file ADVS-13-e20326-s002.docx]

**Supporting Information for**

**Gut microbe-driven Resistance Mechanisms in *Propylea japonica*: Insights from Horizontal Gene Transfer and Oxidative Phosphorylation**

Ningbo HuangFu ^1,2,3^, Xiangzhen Zhu^1,4^, Zhijuan Tang^1,5^, Li Wang^1,4^, Kaixin Zhang^1,4^, Dongyang Li^1,4^, Jichao Ji^1,4^, Jinjie Cui^1,4,^*, Zhaojiang Guo^6,^*, Junyu Luo^1,4,^*, and Xueke Gao^1,4,^*.

Corresponding author: Xueke Gao

Email: gaoxueke@caas.cn.

**This file includes:**

Figures S1 to S11


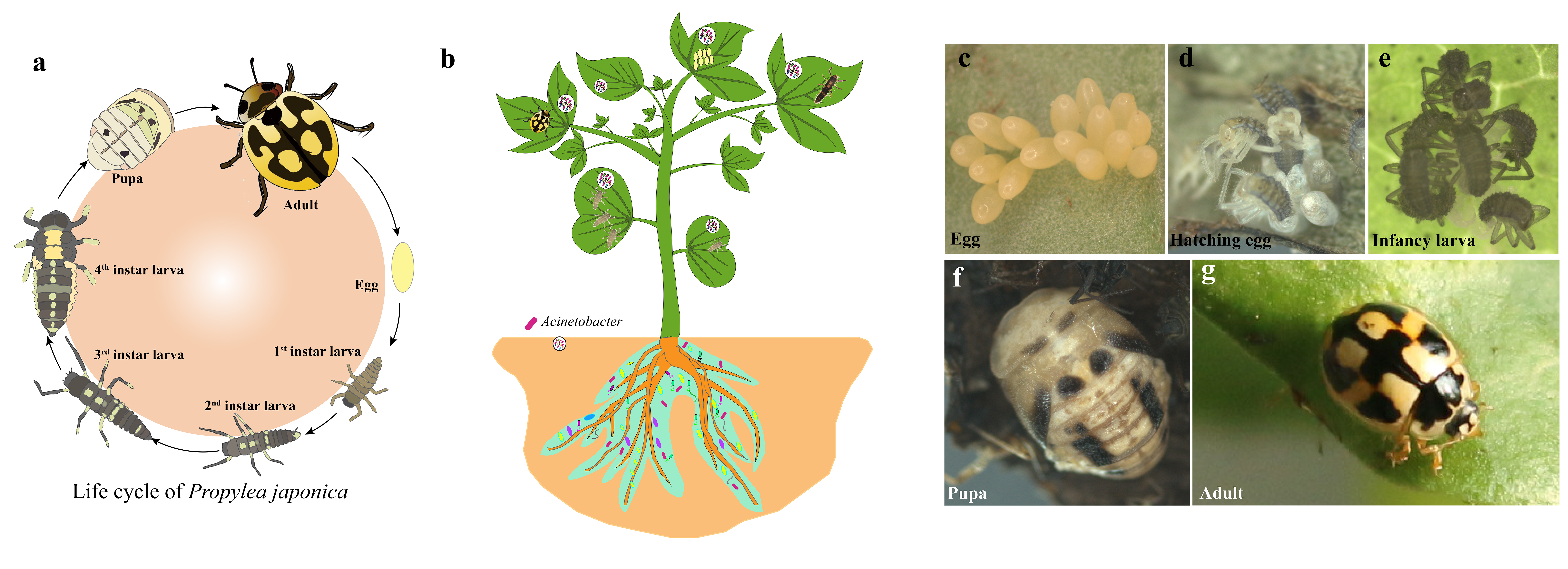


**Supplementary Fig. 1 Life cycle of *Propylea japonica*.** A Schematic overview of ladybird life stages. B Schematic overview of the points at which ladybirds acquire microbes from the environment. C–G Representative images of eggs (C), hatching larvae (D), infant larvae (E), a pupa (F), and an adult (G) reared in the laboratory.


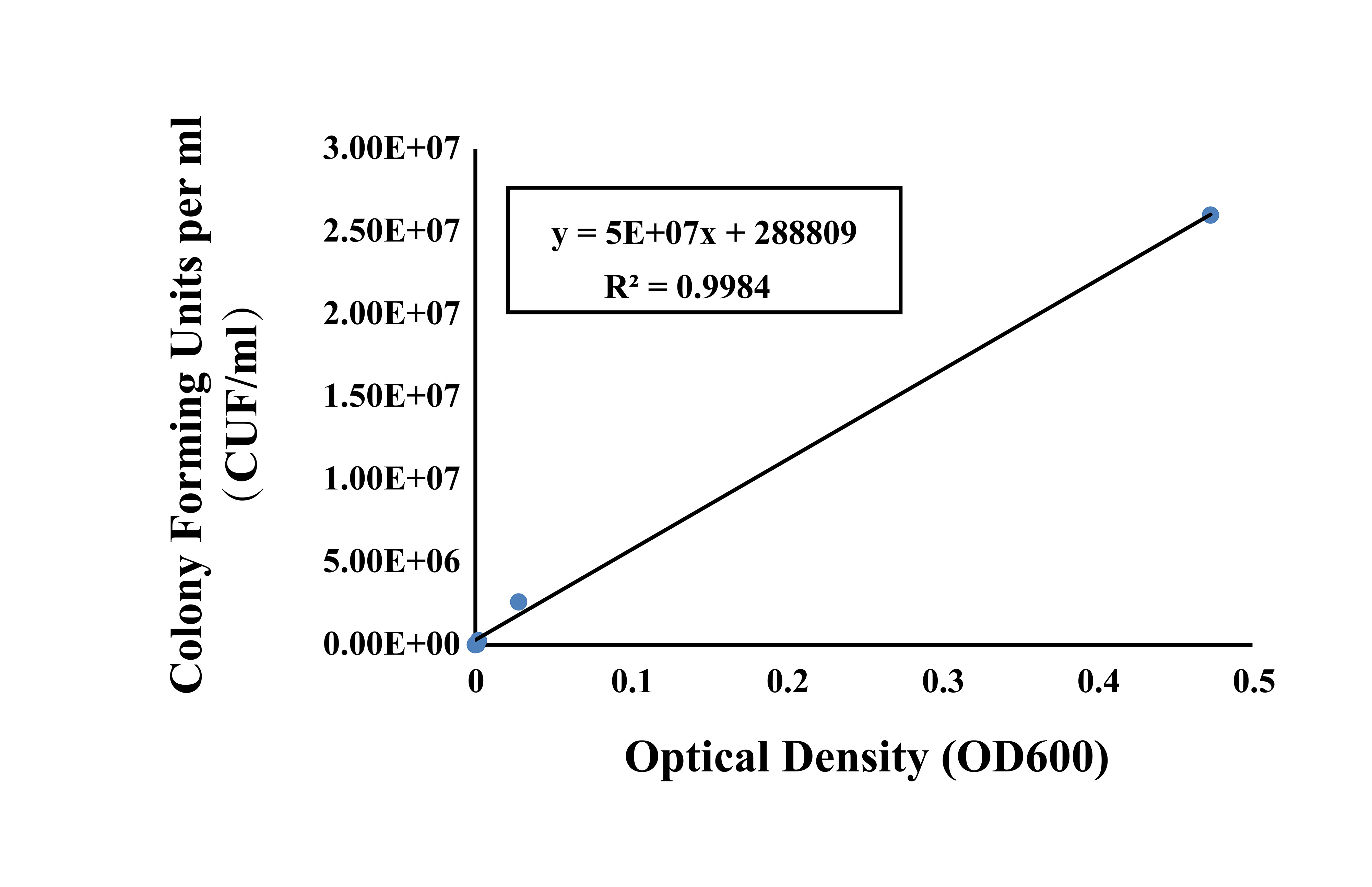


**Supplementary Fig. 2** **The standard curve of bacteria number and OD600 absorbance value.**





**Supplementary Fig. 3 Distribution of *PjDUF1* paralogs across** **coleoptera species.** **A** A total of 53 coleoptera species and 1 outgroup species were selected for the construction of the evolutionary tree. The black box represents the *PjDUF1* and *PjDUF1* paralogs, and the number of black boxes represents the number of homologous genes possessed. The presence (black box) or absence (white box) of *PjDUF1* paralogs were identified by BLASTp search (with ‘‘Expect threshold’’ set at 1E-20). **B** The timeline of speciation and differentiation.


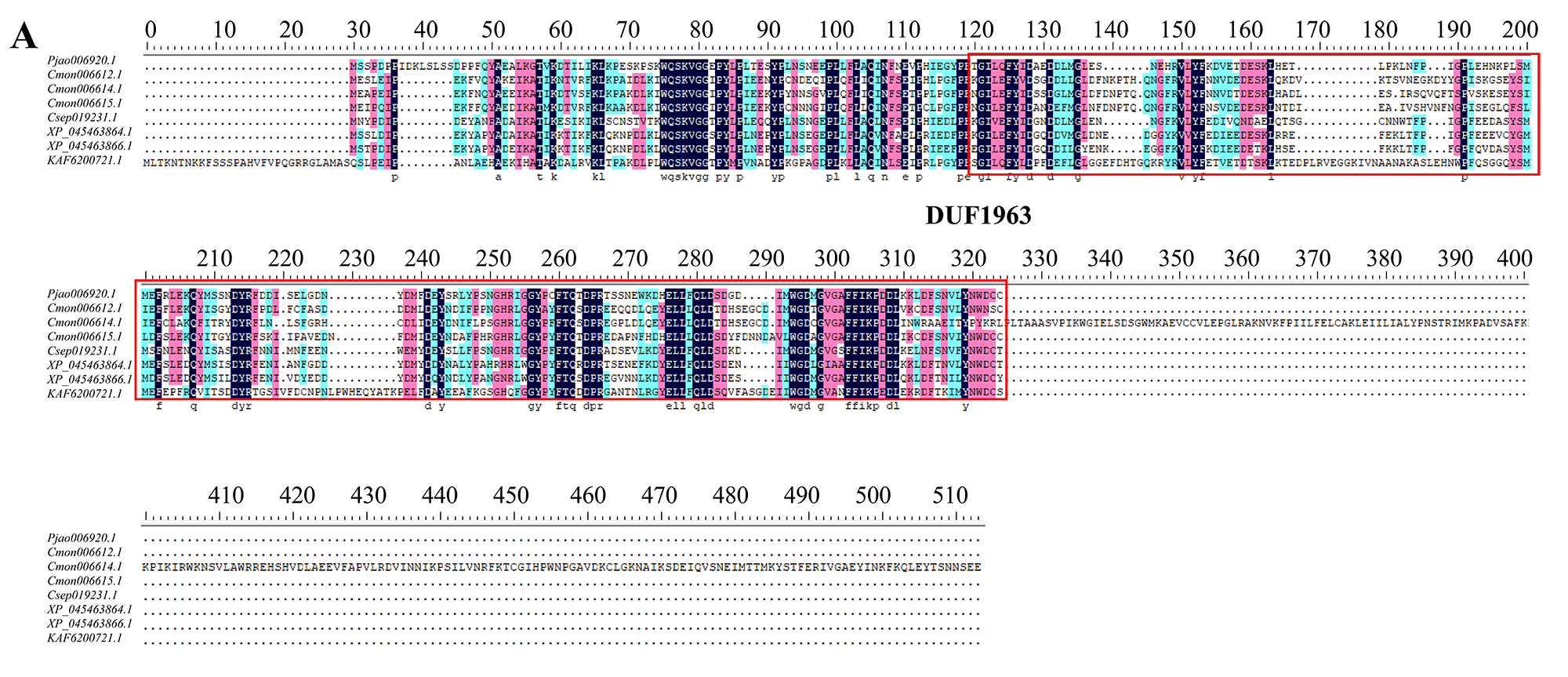


**Supplementary Fig. 4 Alignment of full-length DUF1 amino acid sequences from five insect species.** Red boxes enclose the highly conserved DUF1963 domain.


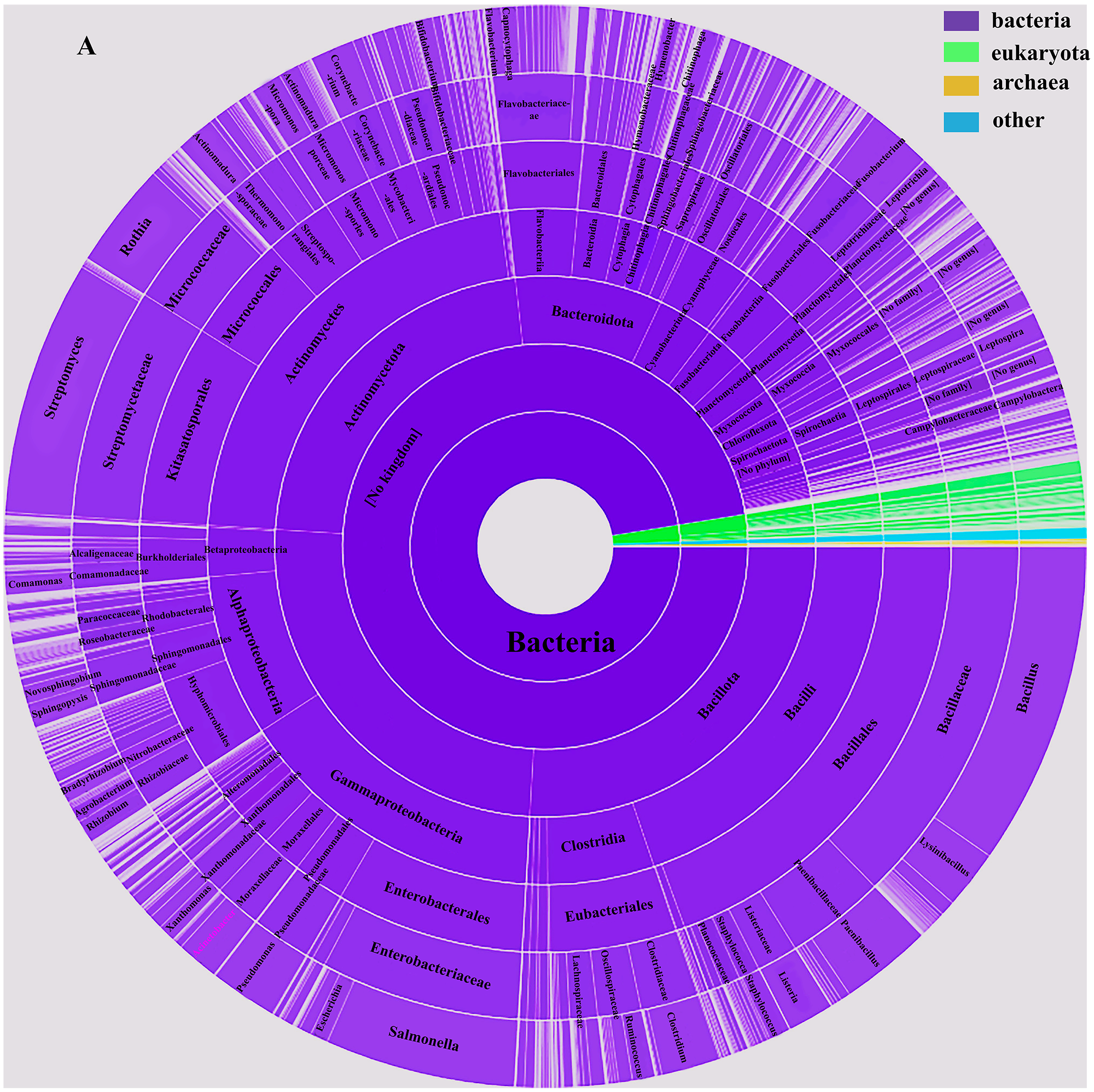


**Supplementary Fig. 5 Distribution of the highly conserved DUF1963 functional domain in 3561 species.** The DUF1963 domain was mainly found in bacteria.


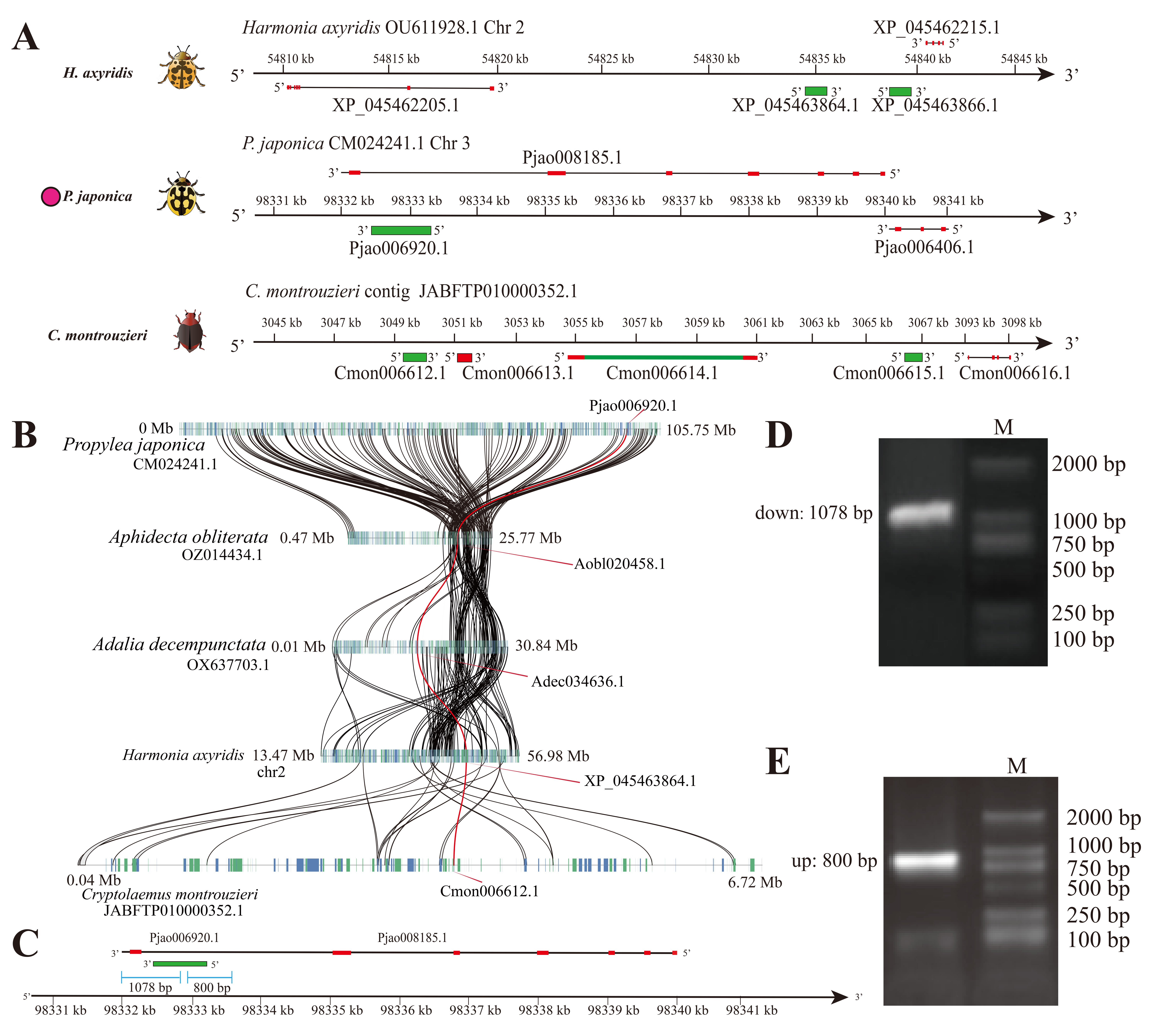


**Supplementary Fig. 6** Evidence for horizontal transfer of *PjDUF1* from bacteria to *P. japonica.* **(A)** Genomic location of *PjDUF1* and its paralogs in predatory ladybirds. **(B)** Synteny analysis of *PjDUF1* genes and its surrounding genes among *P. japonica*, *H. axyridis*, *C. montrouzieri*, *Aphidecta obliterata* and *Adalia decempunctata*. **(C)** Genomic location of *PjDUF1* (Pjao006920.1) in *P. japonica*. Genomic fragments cloned by PCR are indicated in blue. **(D)** Downstream genome fragments (1078 bp) cloned from *P. japonica*. M, marker. **(E)** Upstream genome fragments (800 bp) cloned from *P. japonica*. M, marker.


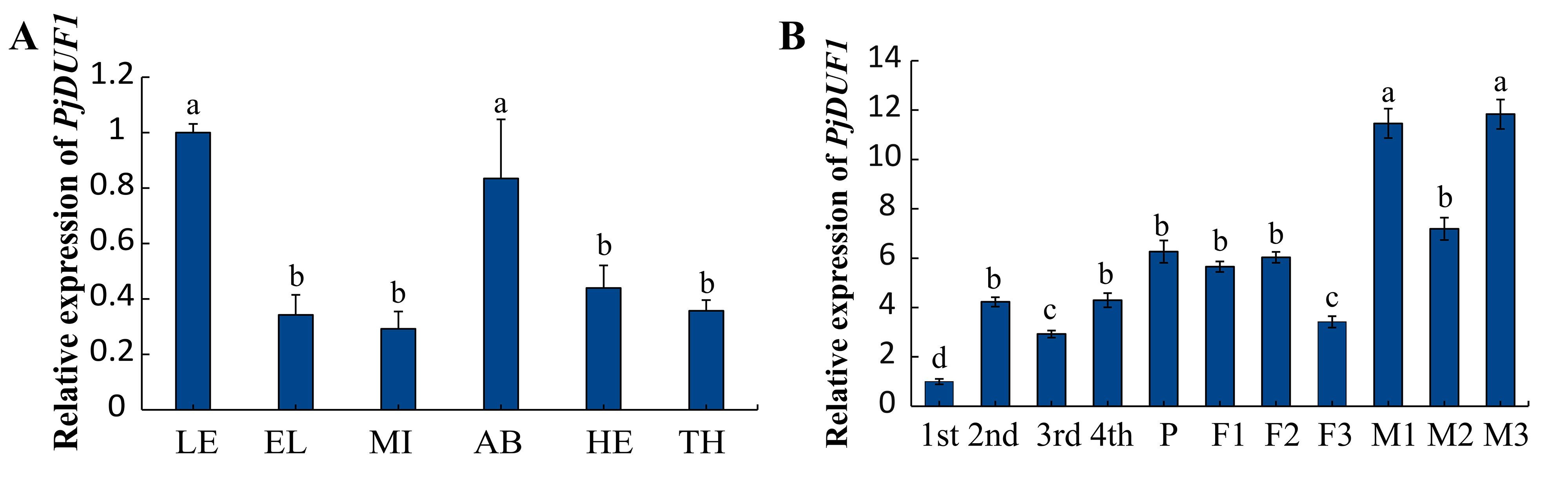


**Supplementary Fig. 7** **Spatiotemporal *PjDUF1* expression profiles.** Spatial expression analysis was conducted by measuring expression levels of *PjDUF1* via quantitative reverse transcription (qRT)-PCR in the leg (LE), elytra (EL), midgut (MI), abdomen (AB), head (HE), and thorax (TH). Temporal expression was analyzed in first, second, third, and fourth instar larvae; in pupae (P); and in male (M) and female (F) adults at days 1, 2, and 3 post-eclosion (PAE). Statistical significance among treatment means was determined by one-way ANOVA followed by Duncan’s multiple range test (p < 0.05); different letters denote significant differences. Data are shown as the mean ± standard error from six biological replicates with three individuals per replicate.


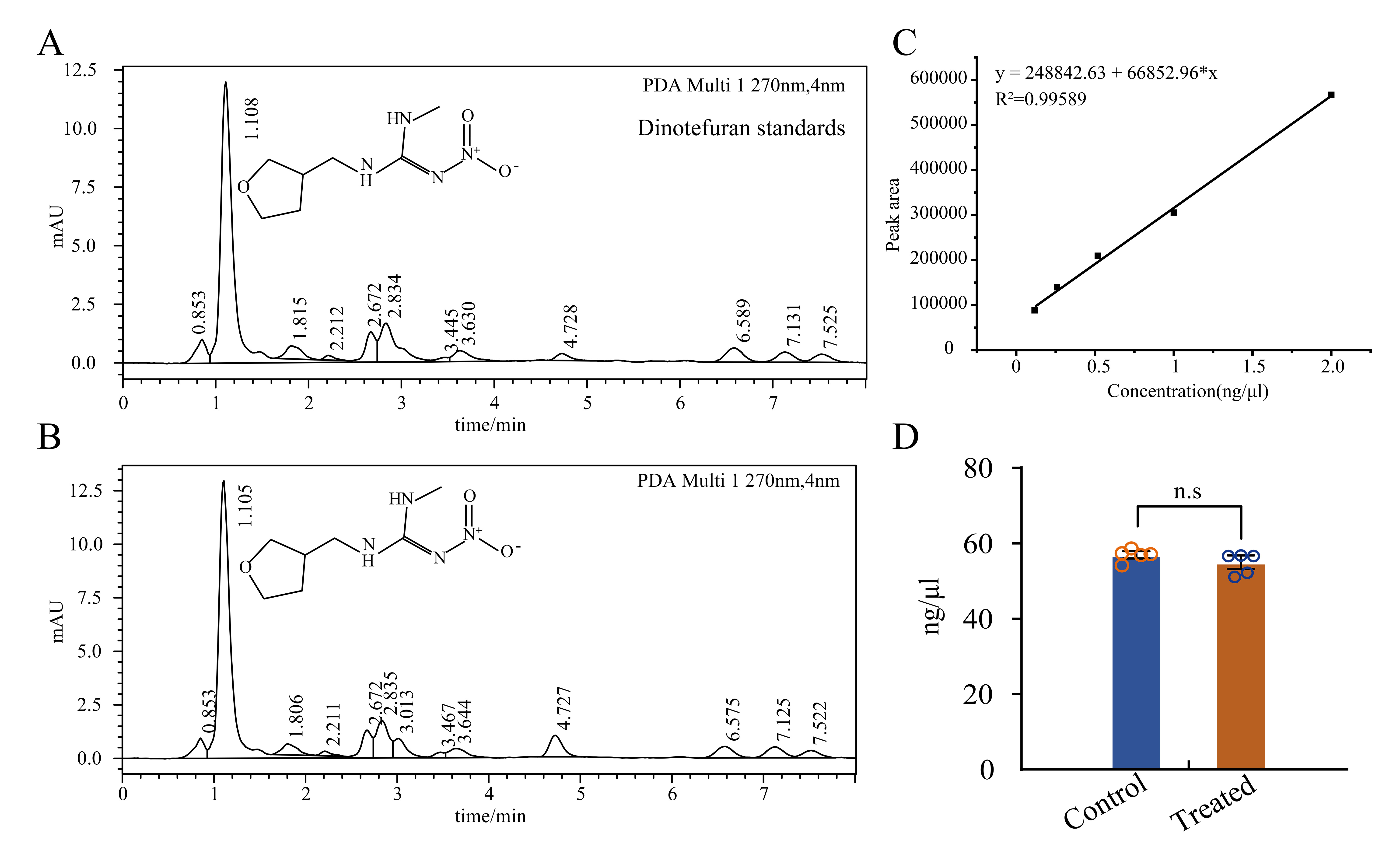


**Supplementary Fig. 8 The detection of substrate dinotefuran level. A** The chromatogram of dinotefuran standards. **B** The chromatogram of dinotefuran in PjDUF1 protein incubation groups. In vitro metabolism studies were performed at 30◦C for 1 h. **C** The liner curve of dinotefuran. **D** The dinotefuran level of PjDUF1 protein incubation groups (treated) and without- PjDUF1 protein incubation groups (Control).





**Supplementary Fig. 9 The effect of *PjDUF1* silencing on the Oxidative phosphorylation signal pathway. A** Newly emerged adult *P. japonica* were injected with 1.5–2 μg PjDUF1-dsRNA. Samples with high interference efficiency were prepared for transcriptomic sequencing. PjDUF1 was confirmed to be downregulated by 80.05% in insects treated with PjDUF1-dsRNA. **B** Expression levels of oxidative phosphorylation pathway genes in different samples. Blue boxes represent significantly downregulated DEGs.


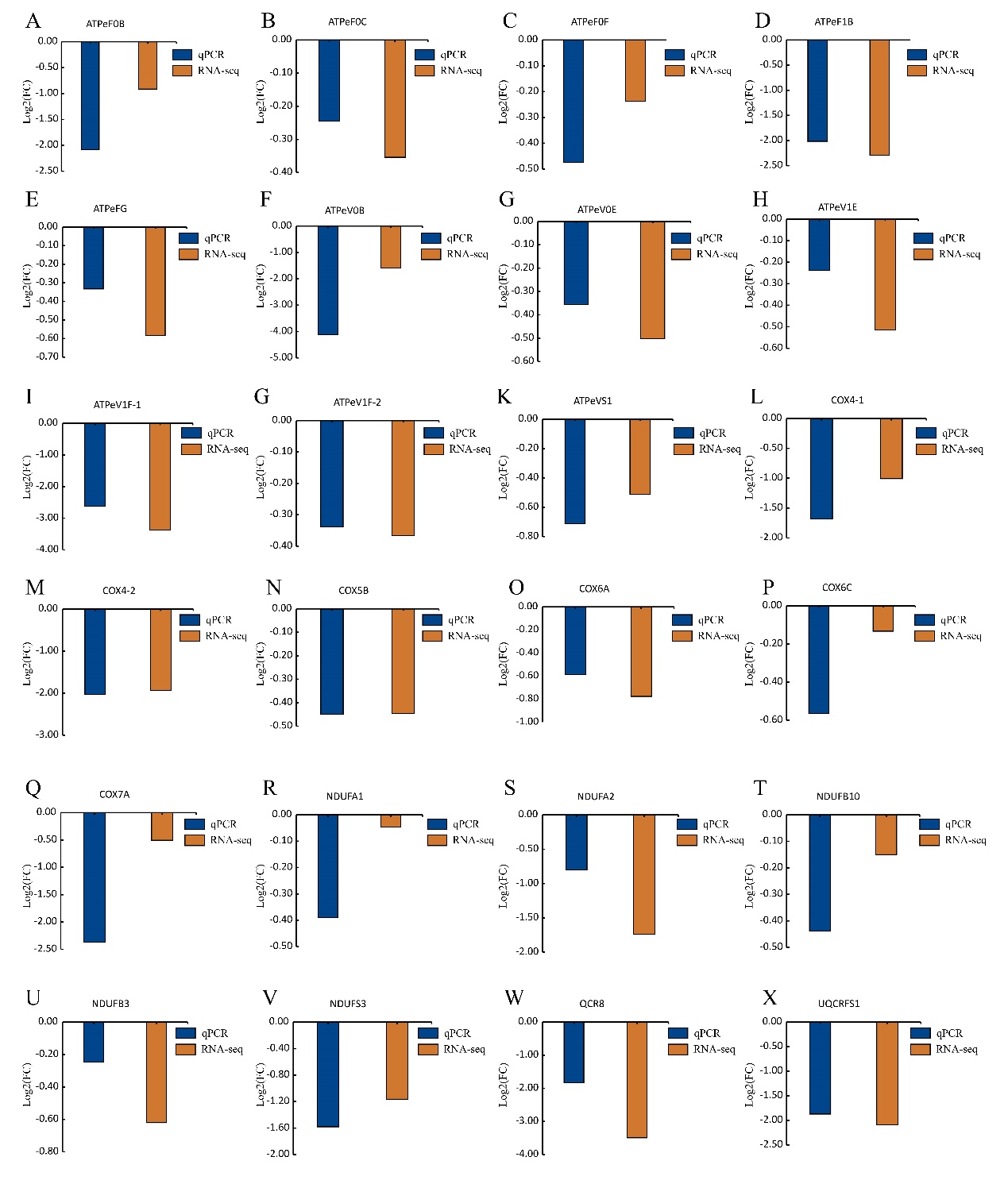


**Supplementary Fig. 10 qRT-PCR of selected DEGs in *P. japonica.*** RNA-seq data were validated with qRT-PCR to measure expression levels of ATP synthase (*ATPeF0B*, *ATPeF0C*, *ATPeF0F-1*, *ATPeF1B*, *ATPeFG*, *ATPeV0B*, *ATPeV0E*, *ATPeV1E*, *ATPeV1F-1*, *ATPeV1F-2*, and *ATPeVS1*), cytochrome c oxidase (*COX4-1*, *COX4-2*, *COX5B*, *COX6A-1*, *COX6C-1*, and *COX7A-1*), NADH dehydrogenase (*NDUFA1*, *NDUFA2*, *NDUFB10*, *NDUFB3*, and *NDUFS3*), and cytochrome c reductase (*QCR8-1* and *UOCRFS1*) genes. Expression levels are shown as the average of six biological replicates with three individuals per replicate.

**
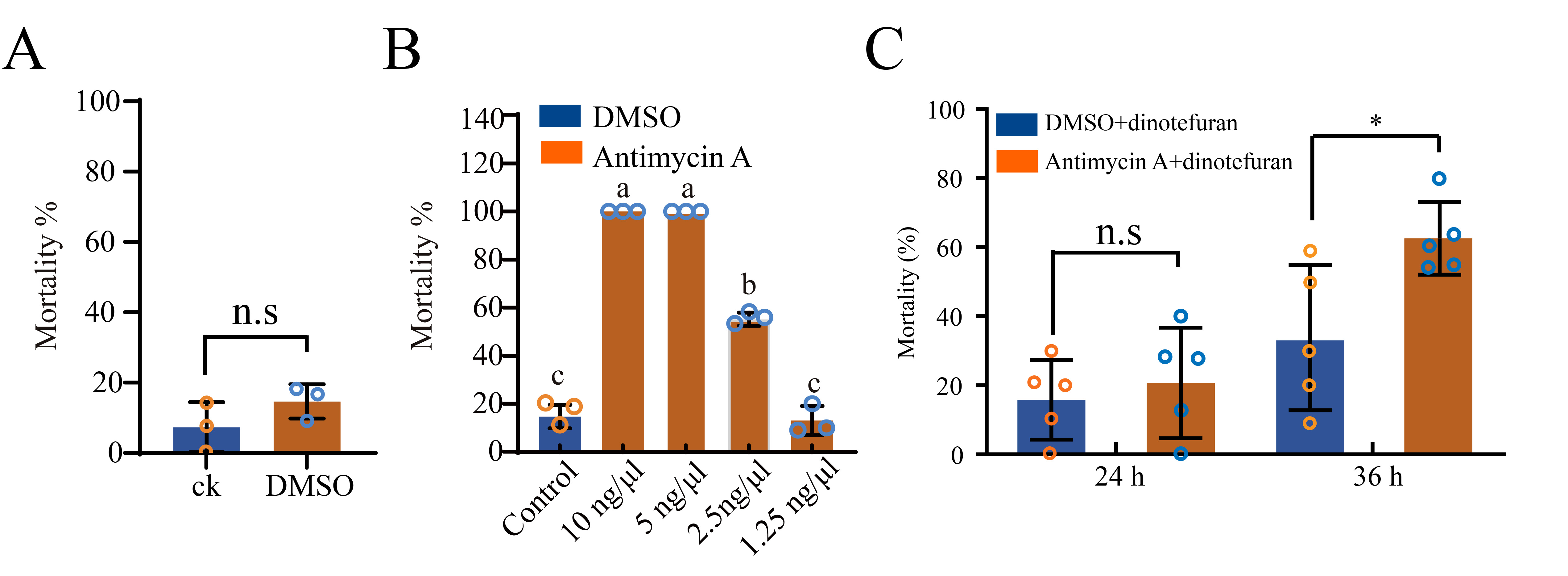
**

**Supplementary Fig. 11 The Effects of Oxidative phosphorylation inhibition on dinotefuran tolerance of *P. japonica*.** **A** The effect of DMSO on the mortality rate of *P. japonica.* **p* < 0.05. n = 3. **B** The effect of different concentrations of antimycin A on the mortality rate of *P. japonica*. **p* < 0.05. n = 3. **C** The Effects of antimycin A on dinotefuran tolerance of *P. japonica.* After 24 hours of treatment with 1.25 ng antimycin A, followed by 1.83 ng dinotefuran treatment. *p < 0.05. n = 5. Statistical significance was determined using one-way ANOVA analysis. All data are represented with mean ± SD.*, p < 0.05; n = 5.
